# Agr2-associated ER stress promotes adherent-invasive *E. coli* dysbiosis and triggers CD103^+^ dendritic cell IL-23-dependent ileocolitis

**DOI:** 10.1016/j.celrep.2022.111637

**Published:** 2022-11-15

**Authors:** Monica Viladomiu, Manirath Khounlotham, Belgin Dogan, Svetlana F. Lima, Ahmed Elsaadi, Emre Cardakli, Jim G. Castellanos, Charles Ng, Jeremy Herzog, Alexi A. Schoenborn, Melissa Ellermann, Bo Liu, Shiying Zhang, Ajay S. Gulati, R. Balfour Sartor, Kenneth W. Simpson, Steven M. Lipkin, Randy S. Longman

**Affiliations:** 1 Department of Medicine, Jill Roberts Institute for Research in Inflammatory Bowel Disease, Weill Cornell Medicine, New York, NY 10021, USA; 2 College of Veterinary Medicine, Cornell University, Ithaca, NY 14853, USA; 3 Department of Pathology, Weill Cornell Medicine, New York, NY 10021, USA; 4 Departments of Medicine and Microbiology and Immunology, Center for Gastrointestinal Biology and Disease, University of North Carolina at Chapel Hill, Chapel Hill, NC 27599, USA; 5 Department of Pediatrics, Center for Gastrointestinal Biology and Disease, University of North Carolina at Chapel Hill, Chapel Hill, NC 27599, USA; 6 Jill Roberts Center for IBD, Division of Gastroenterology and Hepatology, Department of Medicine, Weill Cornell Medicine, New York, NY 10021, USA; 7 Present address: Department of Biological Sciences, University of South Carolina, Columbia, SC 29208, USA; 8 Lead contact

## Abstract

Endoplasmic reticulum (ER) stress is associated with Crohn’s disease (CD), but its impact on host-microbe interaction in disease pathogenesis is not well defined. Functional deficiency in the protein disulfide isomerase anterior gradient 2 (AGR2) has been linked with CD and leads to epithelial cell ER stress and ileocolitis in mice and humans. Here, we show that ileal expression of *AGR2* correlates with mucosal *Enterobactericeae* abundance in human inflammatory bowel disease (IBD) and that *Agr2* deletion leads to ER-stress-dependent expansion of mucosal-associated adherent-invasive *Escherichia coli* (AIEC), which drives Th17 cell ileocolitis in mice. Mechanistically, our data reveal that AIEC-induced epithelial cell ER stress triggers CD103^+^ dendritic cell production of interleukin-23 (IL-23) and that IL-23R is required for ileocolitis in *Agr2*^−/−^ mice. Overall, these data reveal a specific and reciprocal interaction of the expansion of the CD pathobiont AIEC with ER-stress-associated ileocolitis and highlight a distinct cellular mechanism for IL-23-dependent ileocolitis.

## In brief

Viladomiu et al. show that AGR2 is associated with intestinal inflammation, epithelial ER stress, and microbial dysbiosis characterized by the expansion of adherent-invasive *E. coli* (AIEC). The authors identify AIEC as a trigger for ER stress, which is required for Th17 ileocolitis and CD103^+^ DC production of IL-23, and establish the importance of IL-23R signaling for ileocolitis in this model of genetic predisposition to gut inflammation.

## Graphical Abstract

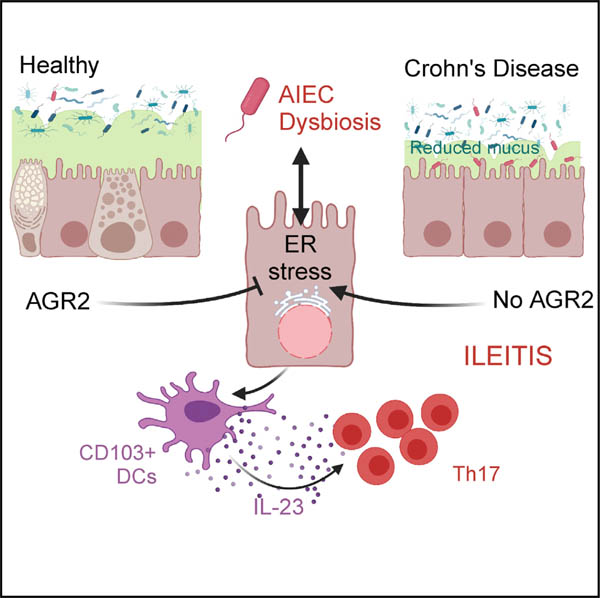

## INTRODUCTION

Crohn’s disease (CD) is a chronic inflammatory disease of the intestine that is characterized by an expansion of mucosal-associated bacteria in genetically susceptible hosts.^1–4^ These adherent bacteria, which include adherent-invasive *Escherichia coli* (AIEC), can serve as pathobionts by triggering immune cell activation;^5–8^ however, the contribution of epithelial signals and CD-associated genetic susceptibility variants that regulate host-microbe interaction pathways are not well defined.

Anterior gradient 2 (AGR2) is a member of the endoplasmic reticulum (ER) protein disulfide isomerase (PDI) family, which ensures proper folding of newly synthesized proteins into their bioactive conformation as they traffic through the ER for subsequent secretion or membrane association.^9–11^ Failure to cope with misfolded protein accumulation leads to ER stress and sub-sequent activation of the unfolded protein response (UPR), a pathophysiological mechanism associated with many inflammatory diseases.^12–16^ *AGR2* is highly expressed in intestinal epithelial cells (IECs), particularly Paneth cells and mucus-producing goblet cells,^17–19^ and is critical for the proper folding and secretion of mucin 2 (MUC2), the major component of the intestinal mucus layer covering IECs along the gastrointestinal tract.^9,20^ Loss-of-function *AGR2* genetic variants have been associated with both CD and ulcerative colitis (UC).^21^ We and others have shown that *Agr2* deletion in mice inhibits intestinal MUC2 synthesis, enhances epithelial ER stress response, and increases inflammatory cytokine secretion.^9,20^ Similarly, a recent report of missense *AGR2* variants in humans revealed reduced goblet cells and increased ER stress in siblings with infantile-onset IBD.^22^

Seminal studies in ER stress and UPR-deficient mice (*Xbp1*^*ΔIEC*^, *Ire1a* ^*ΔIEC*^, *Ire1b*^−/−^, *CHOP*^−/−^, or *MUC2*^−/−^) consistently report a link between intestinal inflammation and bacterial dysbiosis in these models,^12,13,21,23–29^ but the association with particular bacterial taxa is not well defined. While *Agr2*^−/−^ mice in our colony developed severe spontaneous ileocolitis that resembles human inflammatory bowel disease (IBD),^20^ an independently generated *Agr2*^−/−^ mouse model did not develop spontaneous inflammation despite increased susceptibility to experimentally induced acute colitis and a higher incidence of rectal prolapse at very old age.^9^ These results suggest the potential contribution of specific intestinal microbiota to increased severity of intestinal pathology and the development of spontaneous ileitis.

Here, to assess the interaction between AGR2 and CD dysbiosis, we analyzed AGR2-associated microbiome in human IBD and *Agr2*-deficient mice with ileocolitis. Increased ER stress associated with AGR2 correlated with the expansion of mucosal AIEC, which was sufficient to trigger ileocolitis in mono-associated *Agr2*-deficient mice. Our results reveal the selective ability of AIEC to induce ER stress, which is subsequently required for AIEC expansion and production of interleukin-23 (IL-23) by CD103^+^ dendritic cells (DCs) to drive ileocolitis. These results highlight CD103^+^ DCs as a link between AIEC-induced epithelial cell ER stress and ileocolitis in genetically susceptible hosts that may shape therapeutic approaches for CD.

## RESULTS

### AGR2 deficiency correlates with *Enterobacteriaceae* dysbiosis in mouse and human IBD

**Figure 1.**
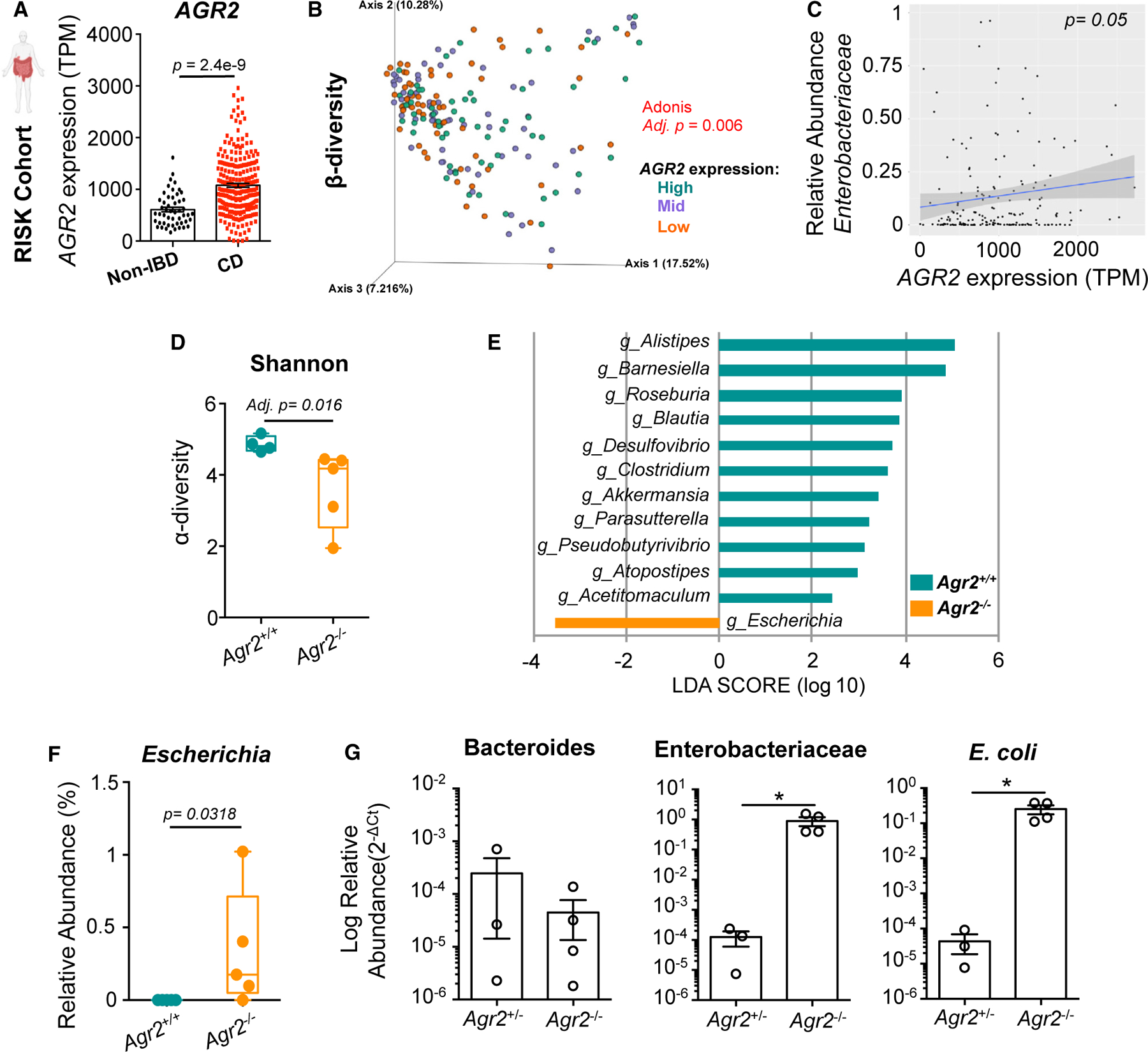
*AGR2* expression correlates with *Enterobacteriaceae* dysbiosis in mouse and human IBD (A–C) Stool 16S sequencing data and matching RNA sequencing from ileal mucosal biopsies were evaluated for patients without IBD (n = 50) and with CD (n = 253) from the RISK Cohort Study. Plot of *AGR2* transcripts per million (TPM) in ileal mucosal biopsies as calculated by Wilcoxon rank-sum test (A). β-diversity between high, mid, and low *AGR2*-expressing individuals was calculated by Bray-Curtis (B). Spearman correlation between mucosal *AGR2* expression and stool *Enterobactericeae* relative abundance (C). (D–F) 16S rRNA sequencing was performed for the stool of littermate *Agr2*^+/+^ and *Agr2*^−/−^ mice (n = 5). α-diversity for 16S rRNA compositional data is shown (D). Linear discriminant analysis effect size (LEfSe) analysis was computed to identify the differentially abundant bacterial genera between *Agr2*^+/+^ and *Agr2*^−/−^ mice. Modules with a linear discriminant analysis (LDA) score >2 are plotted (E). Relative abundance of *Escherichia* genus is shown (F). (G) Relative abundance of *Bacteroides*, *Enterobactericieae*, and *E. coli* normalized to total bacterial 16S rRNA was measured in ileal mucosal scrapings by quantitative PCR. n = 3–4. Error bars represent SEM. *p < 0.05, t test.

CD is characterized by a consistent reduction in taxa diversity and notable alterations in the relative abundance of microbial populations, including a contraction of *Firmicutes* and *Bacteroidetes* and expansion of *Enterobacteriaceae*.^4,30^ To investigate whether *AGR2* is associated with microbial dysbiosis in IBD, we performed an integrated analysis of matched host transcriptome and bacterial 16S rRNA sequencing data in treatment-naive patients with CD from the RISK Cohort Study (Risk Stratification and Identification of Immunogenetic and Microbial Markers of Rapid Disease Progression in Children with Crohn Disease).^1,31^ Patients with CD had higher *AGR2* expression in ileal biopsies compared with non-IBD controls (Figure 1A). 16S rRNA sequencing of the fecal microbiome revealed significant stratification effect of both *AGR2* expression levels and disease diagnosis on β-diversity by linear regression modeling (Figure 1B; Table S1). Spearman correlation between ileal *AGR2* expression and fecal microbiome family relative abundance revealed a positive correlation with *Enterobacteriaceae* (Figure 1C; Table S2).

While *AGR2* expression is higher in patients with IBD in response to increased ER stress and damage, loss-of-function *AGR2* genetic variants have also been associated with both CD and UC for their failure to restrain ER stress.^21^ A recent report of a non-functional *AGR2* missense mutation in siblings with infantile IBD revealed *AGR2* upregulation and increased ER stress.^22^ Therefore, to test whether AGR2 promotes or inhibits *Enterobacteriaceae* dysbiosis, we analyzed the microbiome of littermate, co-housed *Agr2*^−/−^ and *Agr2*^+/+^ mice by 16S rRNA sequencing of ileal contents. Samples were collected from *Agr2*^−/−^ mice with active disease. In our colony, specific-pathogen-free (SPF) *Agr2*^−/−^ mice develop a progressive wasting disease that leads to increased mortality over 4 to 6 months, whereas littermate *Agr2*^+/−^ control mice do not develop any signs of reduced survival, weight loss, or inflammation for up to 8 months (Figures S1A–S1D). Alpha diversity metrics (Shannon index) revealed a contraction in diversity in *Agr2*^−/−^ compared with *Agr2*^+/+^ mice (Figure 1D). Differential taxa and genus relative abundance was assessed using linear discriminant analysis (LDA) effect size (LEfSe).^32^ While 11 bacterial genera were significantly decreased in *Agr2*^−/−^ mice, only *Escherichia* was found to be enriched compared with *Agr2*^+/+^ mice (Figures 1E, 1F, and 1I; Mann-Whitney < 0.05). Quantitative PCR for 16S rRNA in ileal mucosal scrapings revealed a significant expansion of mucosal-associated *Enterobactericieae* and *E. coli* but no differences in *Bacteroides* (Figure 1G).

### AIEC is sufficient to drive microbe-dependent ileocolitis in *Agr2*-deficient mice

**Figure 2.**
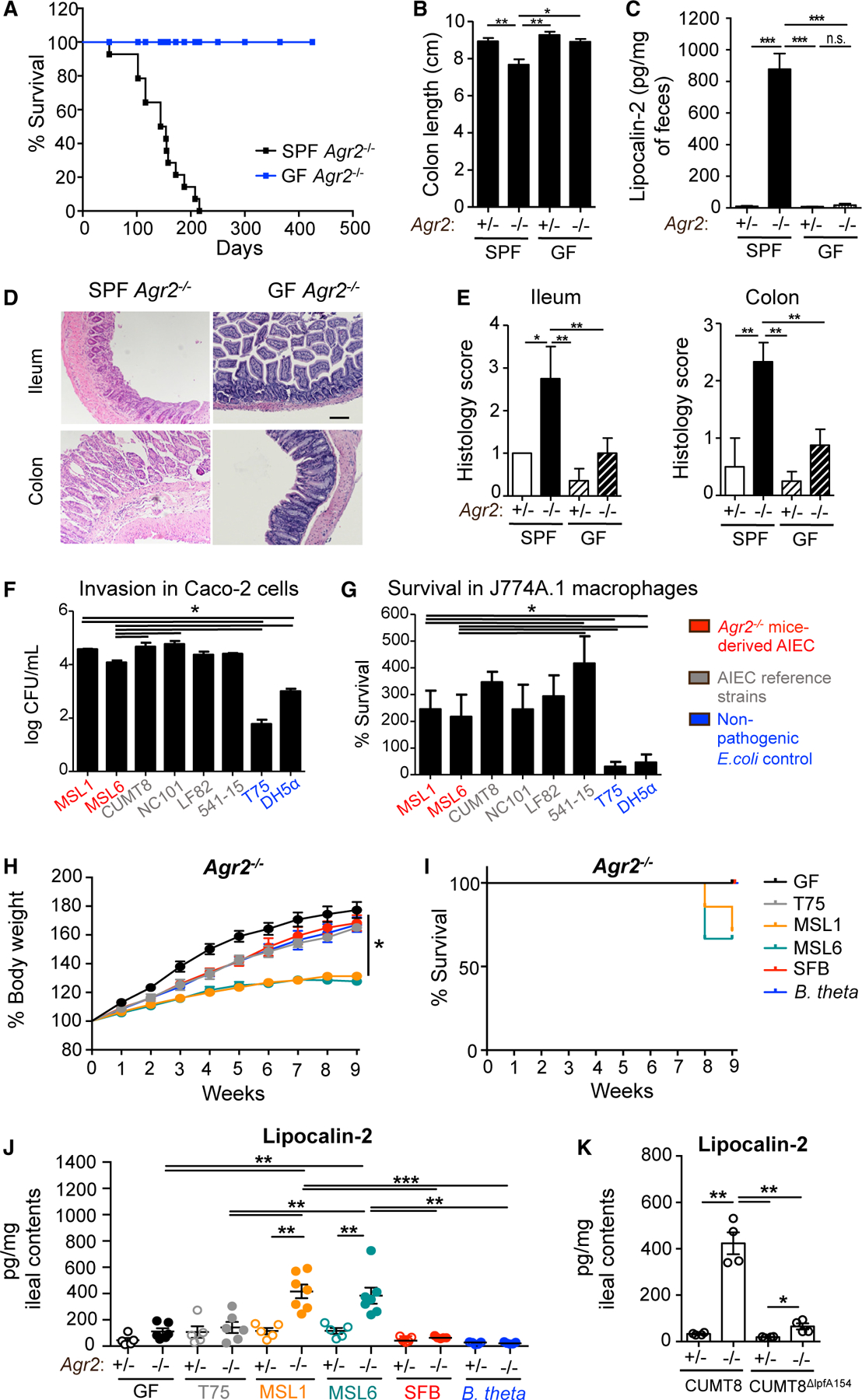
AIEC is sufficient to drive microbe-dependent ileocolitis in *Agr2*-deficient mice (A–E) SPF and GF AGR2-deficient mice were monitored for survival for 14 months (A). Colon length (B) and fecal lipocalin-2 levels (C) were measured at 6 months. Ileum and colon were stained with H&E (D) for histology evaluation (E). n = 4 from one of three experiments. Error bars represent SEM. *p < 0.05, **p < 0.01, ***p < 0.005, t test. Scale bar is 100× mm, 10× magnification. (F and G) Invasion in Caco-2 epithelial cells (F) and survival in J774A.1 macrophages (G) of AIEC isolates. Invasion greater than T75 and DH5-α control values characterize invasive strains; replicating strains show replication values greater than 100%. n = 3 for one of three experiments. Error bars represent SEM. *p < 0.05, Kruskal-Wallis and Dunn’s post-hoc tests. (H–J) GF *Agr2*^+/−^ and *Agr2*^−/−^ mice were mono-colonized with non-pathogenic T75, AIEC MSL1, AIEC MSL6, SFB, or *Bacteroides thetaiotaomicron* for 10 weeks. Mice were monitored for weight gain (H) and survival (I). Lipocalin-2 levels were measured in ileal contents by ELISA (J). n = 4–7 from one of three experiments. Error bars represent SEM. *p < 0.05, **p < 0.01, ***p < 0.005, ANOVA. (K) GF *Agr2*^+/−^ and *Agr2*^−/−^ mice were mono-colonized with AIEC CUMT8 or CUMT8^Δ*lpfA154*^ for 4 weeks. Lipocalin-2 levels were measured in ileal contents by ELISA. n = 4 from one of two experiments. Error bars represent SEM. *p < 0.05, **p < 0.01, ANOVA.

To determine the requirement for gut microbiota in the development of ileocolitis in *Agr2*^−/−^ mice, we re-derived *Agr2*^+/−^ and *Agr2*^−/−^ mice under germ-free (GF) conditions and monitored them for 6 months. While SPF mice developed progressive wasting disease, GF *Agr2*^−/−^ mice had improved survival rates with no death during the period of observation (Figure 2A), no significant colonic shortening (Figure 2B), and no increase in fecal lipocalin-2 levels (Figure 2C) compared with SPF controls. Moreover, they developed significantly less histological inflammation throughout the gastrointestinal tract, particularly in the ileum and colon (Figures 2D and 2E). Consistent with our previous results showing AGR2-dependent Paneth cell abnormalities,^20^ the expression of antimicrobial peptides regenerating family member 3γ (*Reg3*γ) and serum amyloid S1 (*SAA1*) were significantly reduced in the absence of AGR2, and this dysregulation required SPF-associated microbiota (Figure S1E).

Clinical cohorts consistently exhibit the expansion of *Entero-bacteriaceae* in active CD,^1,33–35^ including a unique pathotype of mucosa-associated *E. coli* designated as AIEC.^36,37^ To determine the pathotype of mucosal-associated *E. coli* in *Agr2*^−/−^ mice, we cultured *E. coli* isolates from the ileal mucosa and determined their genotype (random amplified polymorphic DNA [RAPD]-PCR), phylogroup, and virulence gene content. We identified 2 strains (MSL1 and MSL6) that belong to the B2 phylogroup and share many AIEC virulence-associated genes, including *ratA*, *colV*, *kpsMII*, *fyuA*, *malX*, *GSP*, and *iroN* (Figure S2A). Functional analysis of MSL1 and MSL6 isolates revealed invasion of Caco-2 mono-layers (Figure 2F) and persistence within J774A.1 macrophages *in vitro* (Figure 2G), consistent with the AIEC pathotype. Hierarchical clustering analysis showed that both isolates cluster with phylogroup B2 mouse (NC101) and CD-derived (LF82) AIEC isolates compared with mouse (DH5α) and CD-derived (T75) non-AIEC controls (Figure S2B).

To test the possibility that the expansion of mucosal associated AIEC in the ileum of AGR2-deficient mice plays a causal role in spontaneous ileocolitis, GF *Agr2*^+/−^ and *Agr2*^−/−^ mice were mono-colonized at weaning with AIEC MSL1 or AIEC MSL6. Non-pathogenic *E. coli* T75, segmented filamentous bacteria (SFB), and *Bacteroides thetaiotaomicron* (*B. theta*) were used as controls. AIEC-colonized *Agr2*^−/−^ mice showed a growth delay and decreased survival beginning at 8 weeks after colonization compared with non-colonized or T75-, SFB-, and *B. theta*-colonized mice (Figures 2H and 2I), whereas no growth defect or mortality was observed in *Agr2*^+/−^ mice regardless of colonization (Figure S3A). As an indicator of intestinal inflammation, lipocalin-2 levels were increased in the ileal contents of AIEC-colonized *Agr2*^−/−^ mice (Figure 2J). Similar to previous observations,^9^ *Agr2*^−/−^ mice had Paneth cell abnormalities (characterized by larger granules and reduced antimicrobial peptides [AMPs] despite no differences in cell numbers) and reduced goblet cells in the intestinal mucosa (Figures S3B–S3E), which were apparent with both disease-associated AIEC as well as non-invasive *E. coli* colonization. Colonization of *Agr2*^−/−^ mice with B1 phylogroup AIEC CUMT8 was also sufficient to induce inflammation measured by fecal lipocalin-2 by week 4 of colonization compared to littermate controls (Figure 2K). To test whether increased invasion and translocation^3^ contributed to AIEC-induced inflammation, we used a *long polar fimbriae* (*lpfA) 154*-deficient CUMT8 mutant. Unlike CUMT8, colonization of *Agr2*^−/−^ mice with CUMT8^Δ*lpfA154*^ failed to induce inflammation by week 4 (Figure 2K).

### AIEC induce epithelial cell ER-stress-dependent ileocolitis

**Figure 3.**
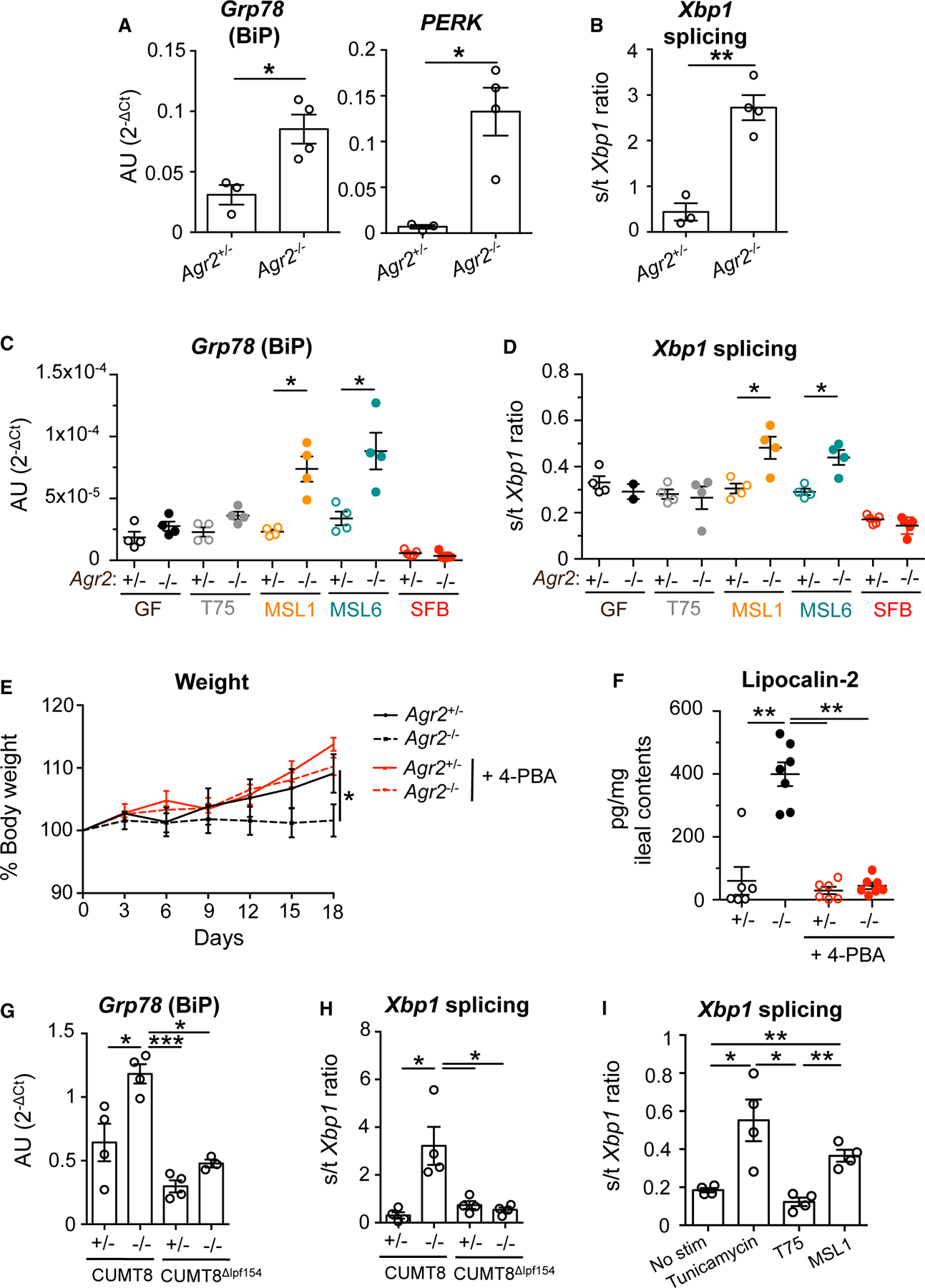
AIEC drive ER-stress-dependent ileocolitis (A and B) Expression of *Grp78* and *Perk* (A) and *Xbp1* splicing (B) were measured by quantitative PCR in ileal epithelial cells of SPF *Agr2*^+/−^ and *Agr2*^−/−^ mice at 6 months. n = 4 from one of two experiments. Error bars represent SEM. *p < 0.05, **p < 0.01, t test. (C and D) GF *Agr2*^+/−^ and *Agr2*^−/−^ mice were mono-colonized at weaning with non-pathogenic T75, AIEC MSL1, AIEC MSL6, or SFB for 10 weeks. *Grp78* expression (C) and *Xbp1* splicing (D) were measured in ileal epithelial cells by quantitative PCR. n = 4 from one of three experiments. Error bars represent SEM. *p < 0.05, ANOVA. (E and F) GF *Agr2*^+/−^ and *Agr2*^−/−^ mice were mono-colonized at weaning with AIEC MSL1 for 3 weeks. Mice were treated with the ER stress inhibitor 4-phenylbutyrate (4-PBA) or PBS control every 3 days. Mice were monitored for body weight gain (E), and lipocalin-2 levels were measured in ileal contents by ELISA (F). n = 6–7 mice per group from one of two experiments. Error bars represent SEM. *p < 0.05, **p < 0.01, ***p < 0.005, ANOVA. (G and H) GF *Agr2*^+/−^ and *Agr2*^−/−^ mice were mono-colonized at weaning with AIEC CUMT8 or CUMT8^Δ*lpfA154*^ for 4 weeks. *Grp78* expression (G) and *Xbp1* splicing (H) were measured in ileal epithelial cells by quantitative PCR. n = 4 mice per group EM. *p < 0.05, **p < 0.01, ***p < 0.005, ANOVA. Organoids were generated from *Agr2*^−/−^ mice. After 1 week, organoid fragments were incubated with AIEC MSL1 or non-pathogenic T75 for 1 h before re-embedding into matrix for new organoid formation. Cells were collected at 48 h, and *Xbp1* splicing was measured by quantitative PCR. n = 4 per group from two of three experiments. Error bars represent SEM. *p < 0.05, **p < 0.01, ANOVA.

Ileal epithelial cells from *Agr2*^−/−^ mice with active disease have increased ER stress as measured by higher *Xbp1* splicing (spliced over total transcripts), BiP expression (a master regulator of the UPR response that reduces ER stress by enhancing cellular folding capacity^38^), and *Perk* expression (a UPR protein activator) compared with heterozygous littermates (Figures 3A and 3B).^9,20^ To evaluate the specificity by which AIEC, but not other commensals, trigger ileocolitis, we investigated whether AIEC differentially regulates AGR2-dependent ER stress in ileal epithelial cells from mono-colonized mice. Consistent with increased ER stress in SPF AGR2-deficient susceptible hosts, increased *Xbp1* splicing and higher expression of *Grp78*, *Perk*, and *Chop* were detected in AIEC-mono-colonized, but not non-pathogenic T75- or SFB-colonized, *Agr2*^−/−^ mice compared with littermate controls (Figures 3C, 3D, and S4A). To test whether ER stress is required for the development of ileocolitis, AIEC MSL1-colonized *Agr2*^+/−^ and *Agr2*^−/−^ mice were treated with ER stress inhibitor 4-phenylbutyrate (4-PBA) for 3 weeks. Decreased transcript levels of *Grp78*, *Perk*, and *Chop* as well as reduced *Xbp1* splicing were observed upon 4-PBA treatment (Figures S4B and S4C), confirming ER stress blockade. 4-PBA-treated MSL1-colonized *Agr2*^−/−^ mice showed no growth delay (Figure 3E) and had decreased lipocalin-2 levels (Figure 3F) compared with untreated *Agr2*^−/−^ controls. Consistent with a requirement for invasive *E. coli*, AIEC CUMT8^Δ*lpfA145*^ mutant failed to induce *Gpr78* expression and *Xbp1* splicing (Figures 3G and 3H).

Consistent with previous work, *Agr2* was highly expressed in mouse epithelial cells compared with lamina propria mononu-clear cells (Figure S4D). Furthermore, consistent with the human IBD data (Figure 1A),^22^ chemical-induced colitis enhanced *Agr2* expression in mice specifically in the epithelial cell compartment (Figure S4D). To determine if AIEC induction of ER stress was epithelial-cell intrinsic, we generated *Agr2*^−/−^ organoids. Organoids exposed to AIEC MSL1, but not non-pathogenic T75, had increased *Xbp1* splicing (Figure 3I) consistent with a direct effect of invasive AIEC on epithelial cells.

### Epithelial cell ER stress promotes AIEC expansion

**Figure 4.**
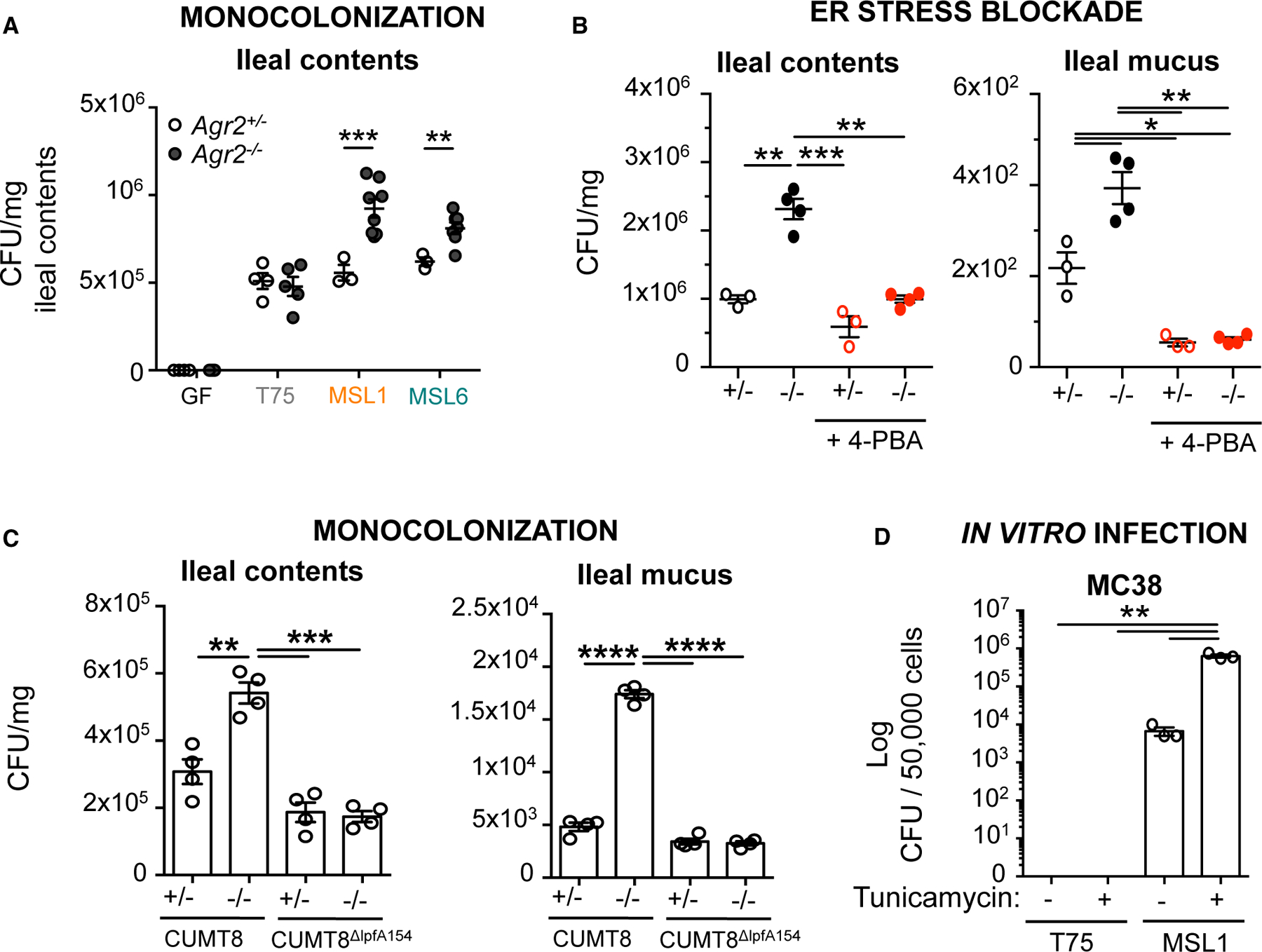
ER stress promotes AIEC expansion GF *Agr2*^+/−^ and *Agr2*^−/−^ mice were mono-colonized at weaning with non-pathogenic T75, AIEC MSL1, or AIEC MSL6 for 10 weeks. Bacterial re-isolation from ileal contents was measured by serial dilution plating. n = 4–7 from one of three experiments. Error bars represent SEM. *p < 0.05, **p < 0.01, ***p < 0.005, ANOVA. (B) GF *Agr2*^+/−^ and *Agr2*^−/−^ mice were mono-colonized at weaning with AIEC MSL1 for 3 weeks. Mice were treated with the ER stress inhibitor 4-PBA or PBS control every 3 days. Bacterial re-isolation from ileal contents and mucus scrapings was measured by serial dilution plating. n = 3–4 mice per group from one of two experiments. Error bars represent SEM. *p < 0.05, **p < 0.01, ***p < 0.005, ANOVA. (C) GF *Agr2*^+/−^ and *Agr2*^−/−^ mice were mono-colonized at weaning with AIEC CUMT8 or CUMT8^Δ*lpfA154*^ for 4 weeks. Bacterial re-isolation from ileal contents and mucus scrapings was measured by serial dilution plating. n = 4 from one of two experiments. Error bars represent SEM. *p < 0.05, **p < 0.01, ***p < 0.005, ****p < 0.001, ANOVA. (D) MC38 epithelial cells were infected for 1 h with AIEC MSL1 or non-pathogenic T75 control following a 4 h tunicamycin treatment for ER stress induction. Bacterial re-isolation was measured by serial dilution plating 24 h post-infection. n = 3 per group from one of three experiments. Error bars represent SEM.*p < 0.05, **p < 0.01, ANOVA.

Surprisingly, increased bacterial loads (colony-forming unit [CFU]/mg) of AIEC MSL1 and MSL6, but not non-pathogenic comparator T75, were detected in the ileal contents of *Agr2*^−/−^ mice compared with their *Agr2*^+/−^ littermates (Figure 4A). While both reduced mucus and AMPs may contribute to the expansion of AIEC, ER stress blockade with 4-PBA significantly reduced AIEC expansion in both ileal contents and mucosa (Figure 4B) without restoring expression of AMPs (Figure S5A) or affecting AIEC growth and infectivity (Figures S5B and S5C). Similarly, mono-colonization with AIEC CUMT8 resulted in higher bacterial loads in ileal contents and mucosal scrapings, while CUMT8 ^Δ*lpfA145*^ mutant did not, suggesting that invasion is critical for the expansion of mucosa-associated AIEC in these susceptible hosts (Figure 4C).

Previous studies in *Salmonella* suggest that epithelial cell ER stress promotes bacterial replication.^39^ To directly test whether epithelial cell ER stress allows for AIEC expansion, we induced ER stress in MC38 epithelial cells with tunicamycin prior to infection with non-pathogenic T75 or AIEC MSL1. Tunicamycin pre-treatment resulted in increased AIEC MSL1 replication compared with non-tunicamycin and T75-infected controls (Figure 4D). These data support a model in which invasive AIEC, but not other mucosal-associated commensals, induce AGR2-dependent ER stress, leading to a feedforward expansion of mucosal-associated AIEC, dysbiosis, and ileocolitis.

### AIEC induces ER stress-dependent Th17 cell ileocolitis in *Agr2*-deficient mice

**Figure 5.**
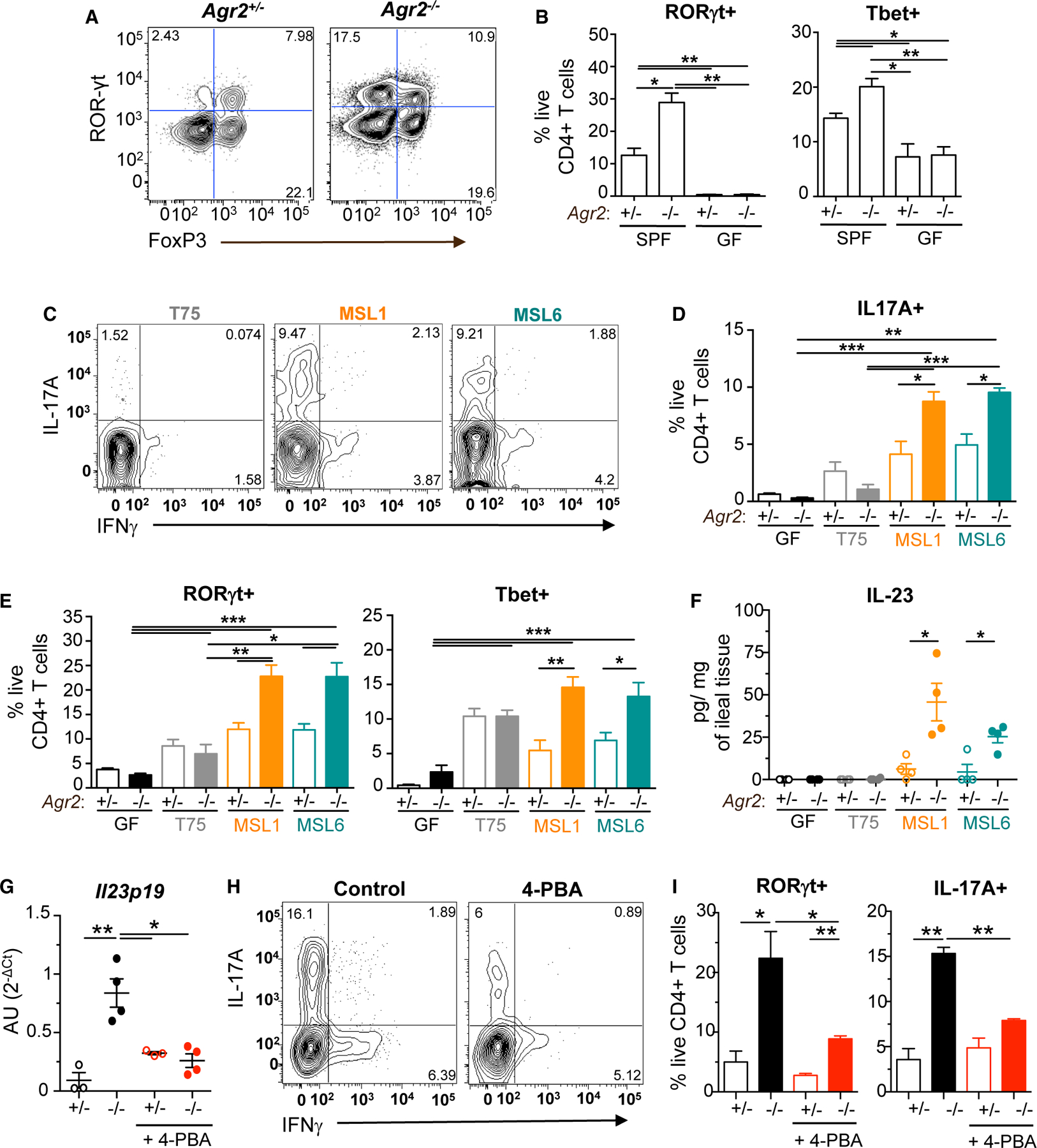
AIEC induces ER-stress-dependent Th17 cell ileocolitis in gnotobiotic *Agr2*^−/−^ mice (A and B) SPF and GF *Agr2*^+/−^ and *Agr2*^−/−^ mice were monitored for 6 months. Flow cytometry of live, CD4^+^ T cells was used to evaluate RORγt and FOXP3 expression in ileal lamina propria (A). Percentage of RORγt^+^ and T-bet^+^ CD4+ T cells was evaluated (B). n = 4 from one of three experiments. Error bars represent SEM. *p < 0.05, **p < 0.01, ANOVA. (C–F) GF *Agr2*^+/−^ and *Agr2*^−/−^ mice were mono-colonized at weaning with non-pathogenic T75, AIEC MSL1, or AIEC MSL6 for 10 weeks. Representative flow cytometry of live, CD4^+^ T cells was used to evaluate IL-17A and IFNγ expression in the ileal lamina propria following a 4 h stimulation with PMA/ionomycin and brefeldin A (C). Percentage of ileal IL-17A^+^ (D) and RORγt^+^/T-bet^+^ (E) CD4^+^ T cells was evaluated. IL-23 production in ileal tissue explants was quantified by ELISA (F). n = 4 from one of four experiments. Error bars represent SEM. *p < 0.05, **p < 0.01, ***p < 0.005, ANOVA. (G–I) GF *Agr2*^+/−^ and *Agr2*^−/−^ mice were mono-colonized at weaning with AIEC MSL1 for 3 weeks. Mice were treated with ER stress inhibitor 4-PBA or PBS control every 3 days. Expression of *Il23p19* was measured in ileal LPMCs by quantitative PCR (G). Representative flow cytometry of live, CD4^+^ T cells was used to evaluate IL-17A and IFNγ expression in the ileal lamina propria (H). Percentage of ileal RORγt^+^ and IL-17A^+^ CD4^+^ T cells was evaluated following a 4 h stimulation with PMA/ionomycin and brefeldin A (I). n = 3–7 mice per group from one of two experiments. Error bars represent SEM. *p < 0.05, **p < 0.01, ANOVA.

To determine the impact of the microbiome on the intestinal T cell activation associated with AGR2-dependent ileocolitis, we profiled ileal lamina propria CD4^+^ T cells in SPF *Agr2*^−/−^ mice with ileocolitis. Compared with age-matched SPF littermate *Agr2*^+/−^ and GF *Agr2*^−/−^ controls, SPF *Agr2*^−/−^ mice had increased percentages of CD4^+^ RAR-orphan receptor γt (RORγt)^+^ T cells and CD4^+^ T-bet^+^ T cells (Figures 5A and 5B). Given this increased Th1 and Th17 cell activation observed in SPF *Agr2*^−/−^ intestines, we examined the sufficiency of AIEC MSL1 and MSL6 to induce T cell activation in mono-associated mice. Flow cytometry analysis revealed a robust induction of IL-17A^+^ CD4^+^ T cells in MSL1- and MSL6-colonized *Agr2*^−/−^ mice compared with uninfected and non-pathogenic T75-colonized *Agr2*^−/−^ mice (Figures 5C and 5D). A corresponding increase was also seen in RORγt^+^ and T-bet^+^ CD4^+^ T cells (Figure 5E). Consistent with the finding of Th17 cell activation, AIEC MSL1 and MSL6 induced IL-23 in ileal tissue explants in the absence of AGR2 (Figure 5F).

Given the ability of other mucosal-associated microbiota to induce IL-23 in the absence of ER stress, we tested if 4-PBA inhibition of ER stress reduced IL-23 production. Consistent with reduced inflammation, ER stress blockade resulted in decreased ileal *Il23p19* gene expression and reduced CD4^+^ROR-γt^+^ and CD4^+^IL-17A^+^ T cell expansion (Figures 5G–5I). These data support a model in which AIEC, but not other mucosal-associated commensals, induce ER-stress-dependent production of IL-23 and Th17 ileocolitis in the absence of AGR2.

### AIEC triggers CD103^+^ DCs and IL-23R-dependent ileocolitis in AGR2 deficiency

**Figure 6.**
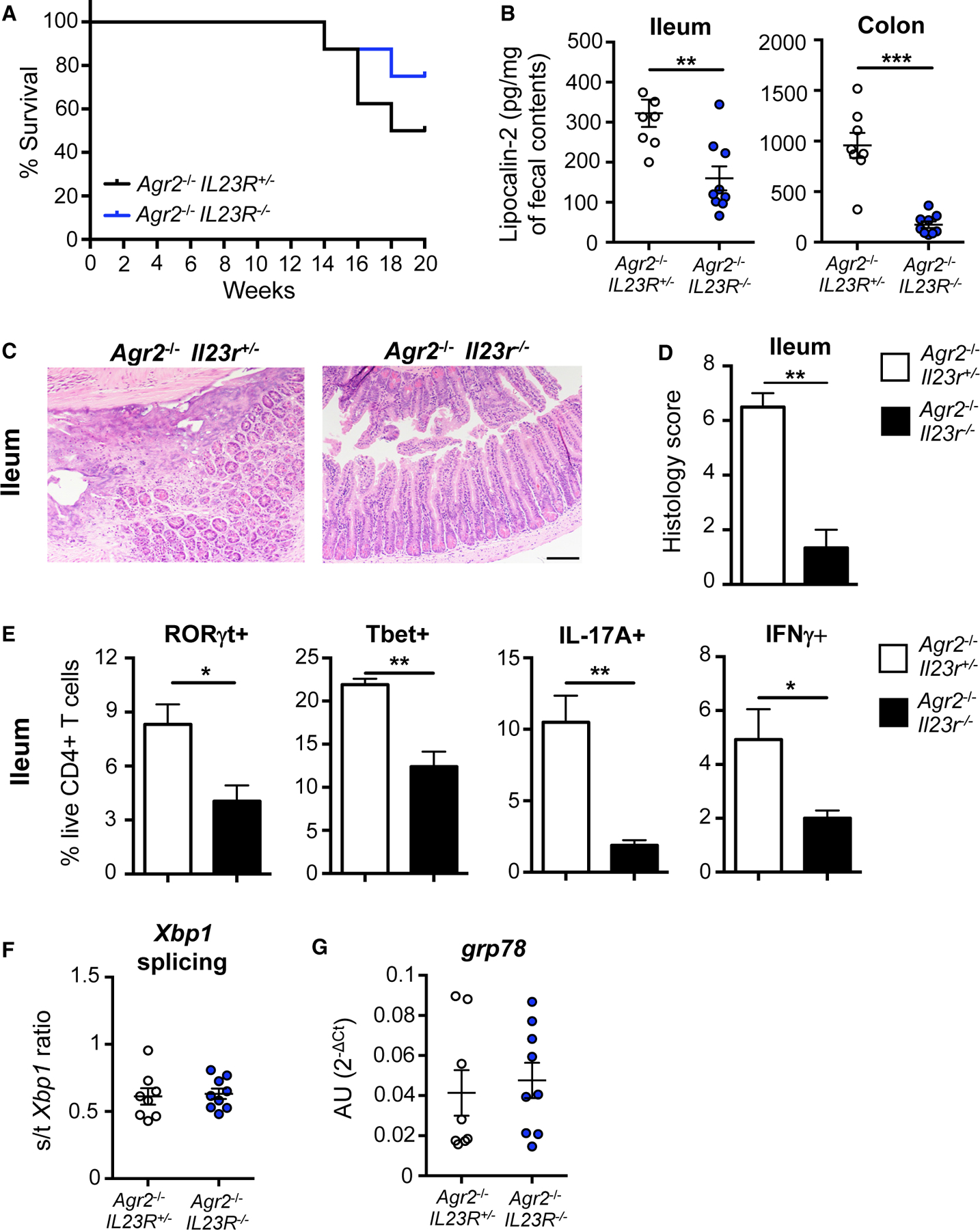
IL-23R is required for ileocolitis in *Agr2*^−/−^ mice (A) SPF *Agr2*^−/−^ *Il23r*^+/−^ and *Agr2*^−/−^ *Il23r*^−/−^ mice were monitored for survival for 20 weeks. (B) Lipocalin-2 levels in ileal and colonic contents were measured by ELISA. (C and D) Ileal sections were stained with H&E (C) for histology evaluation (D). Scale bar, 100 mm, 10×. (E) Percentage of ileal RORgt^+^, T-bet^+^, IL-17A^+^, and IFNγ^+^ CD4^+^ T cells was evaluated by flow cytometry following a 4 h stimulation with PMA/ionomycin and brefeldin A. (F and G) *Xbp1* splicing (F) and *Grp78* expression (G) were measured in ileal epithelial cells by quantitative PCR. n = 8–9. Error bars represent SEM. *p < 0.05, **p < 0.01, t test.

To test whether IL-23 signaling is required to induce disease in *Agr2*^−/−^ mice, we crossed SPF *Agr2*^−/−^ and *Il23r*^−/−^ mice to generate *Agr2*^−/−^ *Il23r*^+/−^ and *Agr2*^−/−^ *Il23r*^−/−^ mice, which were monitored for 20 weeks. IL-23R deficiency delayed mortality in *Agr2*^−/−^ mice (Figure 6A) and reduced lipocalin-2 levels in ileal and colonic contents (Figure 6B). Histological examination of ileal and colonic sections revealed reduced inflammation in *Agr2*^−/−^ *Il23r*^−/−^ mice, characterized by decreased neutrophil infiltration and tissue erosion/ulceration, compared with *Agr2*^−/−^ *Il23r*^+/−^ mice (Figures 6C and 6D). Parallel flow cytometry analysis showed that *Agr2*^−/−^ *Il23r*^−/−^ mice had decreased ileal Th17 (IL-17A and RORγt) and Th1 (interferon [IFN]γ and T-bet) cell expansion (Figure 6E), correlating with disease protection. Despite decreased intestinal inflammation, IL-23R deficiency did not reduce epithelial cell ER stress levels (Figures 6F and 6G).

**Figure 7.**
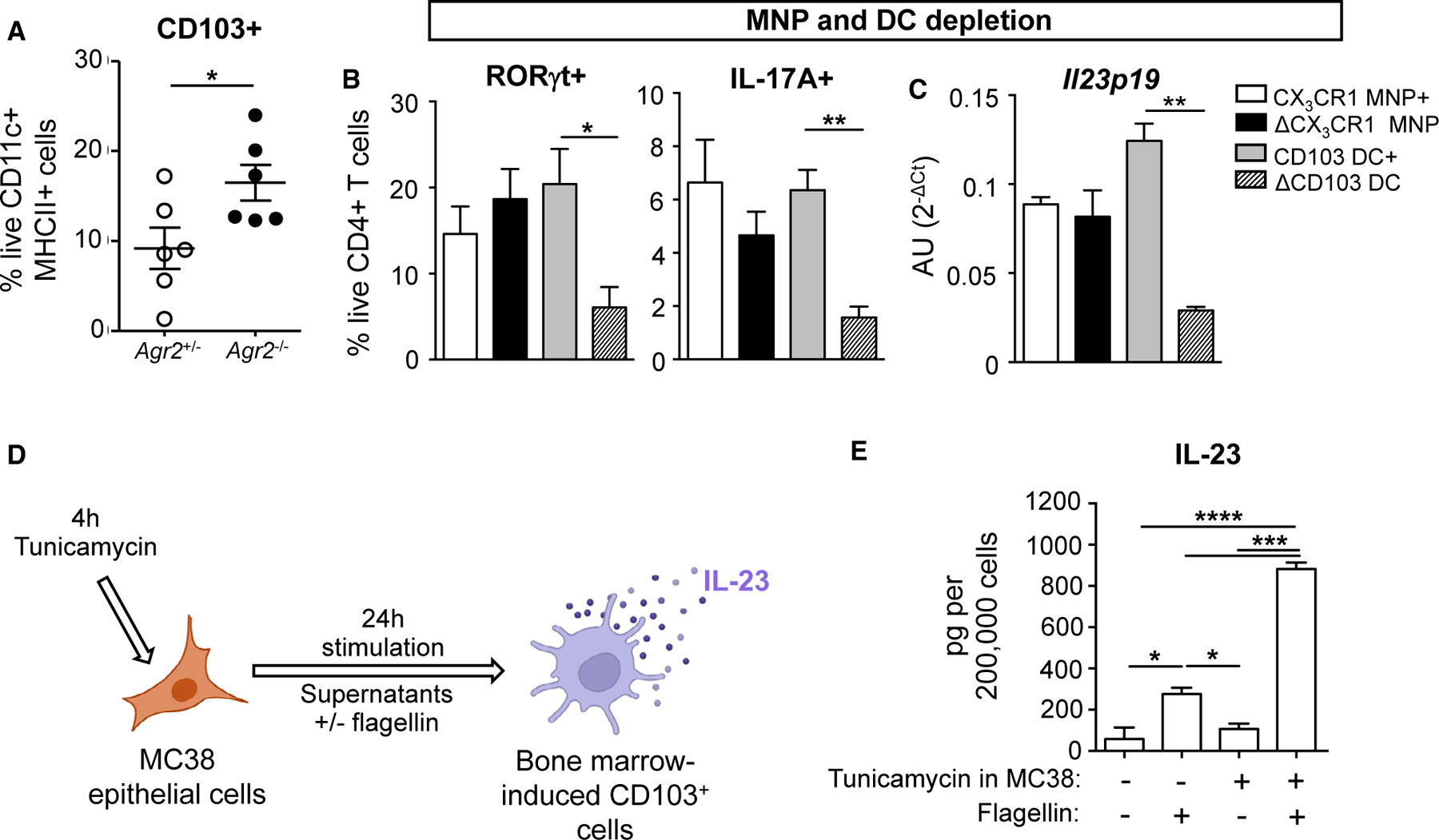
Epithelial cell ER stress triggers CD103^+^ DC production of IL-23 (A) Percentage of live, CD11c^+^major histocompatibility complex (MHC) class II^+^ cells expressing CD103 was evaluated in 6-month-old SPF *Agr2*^+/−^ and *Agr2*^−/−^ mice. n = 6 from one of two experiments. Error bars represent SEM. *p < 0.05, t test. (B and C) 4-week-old littermate CD11c-Cre^−^ CX_3_CR1-STOP-DTR^+/−^ (without DTR, labeled CX_3_CR1 MNP^+^) and CD11c-Cre^+^ CX_3_CR1-STOP-DTR^+/−^ (with DTR, labeled ΔCX_3_CR1 MNP) mice were treated with diphtheria toxin (DT) before and during colonization with 10^10^ CFU AIEC MSL1. Four-week-old littermate langerin-DTR^+/−^ mice were treated with DT (labeled ΔCD103 DC) or PBS (labeled CD103 DC^+^) before and during colonization with AIEC MSL1. Percentage of colonic RORγt^+^ and IL-17A-producing CD4+ T cells was evaluated 10 days after colonization (B). Expression of *Il23p19* was measured in ileal lamina propria cells by quantitative PCR (C). n = 5–6 from one of two experiments. Error bars represent SEM. *p < 0.05, **p < 0.01, ANOVA. (D and E) MC38 cells were treated with 10 μM tunicamycin for 1 h to induce ER stress. Cells were then washed and cultured for 4 h with complete media. After 4 h, MC38 supernatants were collected and used to stimulate bone marrow (BM)-induced CD103^+^ cells in the presence or absence of flagellin for 24 h (D). IL-23 production was measured by ELISA (E). n = 3 from one of two experiments. Error bars represent SEM. *p < 0.05, **p < 0.01, ***p < 0.005, ****p < 0.001, ANOVA.

SPF *Agr2*^−/−^ mice with spontaneous ileocolitis show increased numbers of CD103^+^ DCs in the ileum (Figure 7A). To evaluate if these CD103^+^ DCs were required for the induction of IL-23 and Th17 cells by AIEC isolate MSL1, we utilized genetic mouse models to perform selective depletion of these subsets *in vivo*. Injection of diptheria toxin (DT) was used to selectively deplete CX_3_CR1^+^ mononuclear phagocytes (MNPs) expressing the DT receptor (DTR) using *Itgax-cre Cx3cr1-LSL-DTR* mice^40^ or conventional CD103^+^ DCs using *Lang-DTREGFP* mice^41^ (Figure S6). Following 10 days of colonization with AIEC MSL1, depletion of CD103^+^ DCs resulted in a significant reduction of RORγt^+^ and IL17A-producing CD4^+^ T cells (Figure 7B), while CX_3_CR1^+^ MNP depletion did not impact Th17 responses. Moreover, CD103^+^ DC depletion decreased ileal *il23p19* production (Figure 7C). To assess whether CD103^+^ DCs can sense epithelial ER stress to produce IL-23, we set up an *in vitro* model by which we induced ER stress in epithelial cell line MC38 with tunicamycin. MC38 cell supernatants were then collected and used to stimulate bone-marrow-induced CD103^+^ (iCD103^+^) cells^42^ in the presence or absence of flagellin (Figure 7D). While tunicamycin-treated MC38 supernatants did not activate IL-23 production by iCD103^+^ cells alone, they synergized with flagellin to enhance IL-23 production (Figure 7E). These findings support a model in which CD103^+^ DCs sense AIEC-induced epithelial ER stress and trigger IL-23 dependent ileocolitis.

## DISCUSSION

Genetic variants leading to ER stress and activation of the UPR are associated with inflammatory bowel disease, but the environmental signals required to trigger disease are not well understood. Seminal studies using epithelial-cell-specific UPR-deficient mice, as well as other genetic models targeting ER-associated degradation (ERAD), have revealed a role for ER stress in microbiota-driven intestinal inflammation;^12,13,21,23–29,43,44^ however, the nature and specificity of the pathobionts driving disease are less well defined. Here, we reveal a specific interaction between *AGR2* expression and *Enterobacteriaceae* dysbiosis in human CD. In humans, the correlation of *AGR2* expression with *Enterobacteriaceae* dysbiosis may reflect a compensatory response to increased ER stress triggered during CD or increased expression of non-functional variants.^22^ Supporting this association with increased ER stress, our results in *Agr2*-deficient mice with increased ER stress and spontaneous ileocolitis revealed *Enterobacteriaceae* dysbiosis with specific enrichment of AIEC in the mucosal-associated microbiota. While we cannot rule out the additional contribution of other microbiota, mono-colonization studies performed here reveal that AIEC are sufficient to trigger disease in this genetically susceptible model. The convergent data from mouse models and human CD reveal a specific interaction between AGR2 and microbial dysbiosis and highlight an essential role for the CD-associated pathobiont AIEC as an environmental trigger in ER-stress-dependent disease.

Mucosal adherence and invasion are critical features enabling AIEC-induced inflammation and Th17-dependent colitis, but the signals conferred by epithelial cells are not well defined. Here, we show the specificity of AIEC, but not non-pathogenic *E. coli* or other mucosal-associated commensals such as SFB, to induce epithelial ER stress and subsequent development of ileocolitis in the absence of AGR2. Although the molecular characteristics of the microbial triggers involved in activating the UPR still need to be defined, our *in vitro* studies using *Agr2*-deficient organoids support the sufficiency of AIEC to directly induce epithelial-cell-intrinsic ER stress. While AIEC lacks typical virulence factors, these findings mechanistically highlight epithelial cell ER stress as a critical step by which the CD-associated pathobiont AIEC induces intestinal inflammation.

In addition to triggering downstream inflammation, epithelial cell ER stress reciprocally promotes AIEC dysbiosis. Similar to previous studies in *Salmonella*,^39^ our data reveal the ability of epithelial cell ER stress to promote the expansion of AIEC. Although reduced mucus as well as impaired Paneth cell production of AMPs in AGR2-deficient mice may contribute to higher levels of mucosa-associated *E. coli,* as observed in both SPF and AIEC mono-colonized AGR2-deficient mice, inhibitors of ER stress block AIEC expansion and downstream inflammation without restoring Paneth cell AMP expression. Alternate ER-stress-sensitive functions of secretory cells may still contribute to AIEC dysbiosis. In addition, the induction of ER stress by AIEC may further potentiate the mucosal enrichment of AIEC by inducing the expression of host cell receptors for AIEC outer membrane vesicles (OMVs) such as Gp96 and CEACAM6 on the apical surface of IECs as previously described.^45^ Once inflammation is established, inflammatory metabolites can also support AIEC expansion.^46^ Collectively, these findings highlight an additional contribution of ER stress to ileocolitis by promoting and reinforcing AIEC expansion.

In addition to regulating microbial homeostasis, ER stress pathways induced by AIEC regulate its specific and selective link with inflammatory Th17 cell ileocolitis. Although other bacteria including SFB specifically expand intestinal Th17 cells,^47^ their induction of Th17 immunity has been associated with protective responses against the intestinal pathogen *Citrobacter rodentium*. Here, we show that AIEC, but not SFB, is associated with Th17 responses that exacerbate disease and promote ileocolitis in an AGR2-deficient background, highlighting the requirement for ER stress and potentially other factors in shaping pathogenic Th17 cell activation in this model.^48^ While increased Th17 cells parallels previous observations establishing the connection between AIEC and pathogenic Th17 cell induction in various genetic backgrounds,^5,8,49^ our depletion studies reveal an alternate model in which CD103^+^ DCs regulate IL-23-dependent disease associated with ER stress. While these results contrast with the dependency for CX_3_CR1^+^ MNPs in sensing AIEC-derived propionate and regulating Th17 responses to SFB,^50,51^ they highlight the selective contribution of CD103^+^ DCs to induce IL-23-dependent inflammatory Th17 cells following AIEC-induced ER stress, as similarly described following their phagocytosis of infected apoptotic epithelial cells.^52^ Collectively, these findings reveal a selective and specific role for AIEC in triggering ER stress, which, sensed by a different mechanism than homeostatic commensal Th17 cell induction, drives intestinal inflammation.

Our findings highlight CD103^+^ DCs as a link between AIEC-induced epithelial cell UPR and ileocolitis in genetically susceptible hosts that may shape therapeutic approaches for CD. These results linking AIEC-induced ER stress with IL-23R-dependent disease may also reflect degenerate and/or synergistic pathways seen in other UPR-induced IL-23-dependent diseases.^53^ While our results suggest that microbial-dependent induction of ER stress is upstream of IL-23, they do not exclude potential downstream synergistic effects of IL-23-dependent IL-22 in acute inflammation.^54^ Additional studies are needed to understand the mechanisms by which CD103^+^ DCs sense exogenous ER stress signals and the potential impact of these findings in positioning selective IL-23 blockade for the treatment of CD.

Overall, the results reveal a link between the IBD-associated pathobiont AIEC and epithelial cell ER stress underlying disease pathogenesis. These findings propose a revised model in which microbiota-induced ER stress in genetically susceptible individuals contributes to both a feedforward cycle of dysbiosis as well as downstream inflammation. Future studies are needed to address the potential for these microbial and mucosal biomarkers as targets for stratifying disease pathogenesis and alternate treatment strategies for IBD.

### Limitations of the study

Further studies are needed to elucidate the specific molecular mechanisms by which AIEC triggers epithelial cell ER stress and their interaction with AGR2. Although our results reveal the sufficiency of AIEC, but not other adherent commensals or non-invasive *E. coli*, to induce ER stress and ileocolitis in the absence of AGR2, we do not rule out the ability of additional pathobionts to induce disease. In addition, while inhibitors of ER stress block AIEC expansion and downstream inflammation without restoring Paneth cell AMP expression, alternate ER-stress-responsive functions of secretory cells may still contribute to AIEC dysbiosis and require further evaluation. Lastly, the signals conferred by epithelial cells to promote downstream inflammation are not well defined. Specifically, a mechanistic understanding of how CD103^+^ DCs sense epithelial cell ER stress to trigger IL-23 and shape inflammatory Th17 cell responses has the potential to impact therapeutic approaches for IBD.

## STAR★METHODS

### RESOURCE AVAILABILITY

#### Lead contact

Further information and requests for reagents may be directed to and will be fulfilled by lead contact Randy S. Longman (ral2006@med.cornell.edu).

#### Materials availability

All unique/stable reagents generated in this study are available from the lead contact with a completed Materials Transfer Agreement.

#### Data and code availability

- 16SrRNA sequencing has been deposited at NCBI SRA (BioProject ID PRJNA886765). RISK Cohort data used for analysis is publicly available: RNA sequencing from biopsies (GEO GSE101794) and paired 16SrRNA sequencing from stool samples (SRA Bioproject PRJNA237362).
- This paper does not report original code.
- Any additional information required to reanalyze the data reported in this paper is available from the lead contact upon request.

### EXPERIMENTAL MODEL AND SUBJECT DETAILS

#### Housing and husbandry of experimental animals

*Agr2*^*+/−*^, *Agr2*^−/−^, *Agr2*^−/−^*Il23r*^*+/−*^ and *Agr2*^−/−^*Il23r*^−/−^ mice^20,56^ were bred and housed under specific pathogen free (SPF) conditions at the Research Animal Resource Center vivarium at Weill Cornell School of Medicine (WCM). *Itgax-cre*, *Cx3cr1-LSL-DTR*^40^ *and Lang-DTREGFP*^41^ mice were purchased from The Jackson Laboratory.

GF *Agr2*^−/−^ mice were re-derived at the National Gnotobiotic Rodent Research Center at the University of North Carolina (UNC) at Chapel Hill. Mice were then bred and maintained in sterile, HEPA filter-controlled, isolator cages (Allentown) in the Gnotobiotic Facility at WCM. Samples were collected monthly for culture-based methods to verify GF status.

All mice were group housed with a 12 h light/dark cycle and allowed *ad libitum* access to diet and water. All experiments were performed with 6–8 week old littermates unless specified. Both male and female mice were used with random and equal assignment of same sex to each experimental group. All animal studies were carried out in accordance with protocols approved by the Institutional Animal Care and Use Committee (IACUC) at WCM.

#### Growth and isolation of bacterial strains

AIEC strains MSL1 and MSL6 were isolated from ileal mucosa of *Agr2*^−/−^ mice and cultured as described previously.^37^ Dr. Kenneth Simpson provided the non-pathogenic T75 and DH5α control strains and the AIEC CUMT8, CUMT8^Δ*lpfA154*^, NC101 and LF82 strains. All *E. coli* strains and *Bacteroides thetaiotaomicron*^55^ were grown anaerobically at 37°C in Peptone Yeast Extract Broth with Glucose media (PYG, Anaerobe Systems).

#### Colonization of mice with cultured bacteria

For gnotobiotic experiments, *Agr2*^−/−^ and *Agr2*^*+/*−^ littermate controls were colonized at weaning (21 days old) with a single dose of 2 × 10^9^ CFU log-phase bacteria by sterile oral gavage. For infection of SPF mice, littermate controls were colonized at weaning with a single dose of 10^10^ CFU log-phase bacteria by sterile oral gavage.

### METHOD DETAILS

#### Microbial amplicon sequencing

16S amplicon sequencing was performed at MR DNA (Shallowater, TX) as previously described^58^ using 16S primers. Sequencing was performed on a Roche 454 FLX Titanium instrument following the manufacturer’s instructions.

#### Microbiome analyses

Raw sequence data were processed using proprietary software (www.mrdnalab.com, MR DNA, Shallowater, TX). Specifically, barcodes and primers were trimmed, after which sequences shorter than 200bp were removed, as well as sequences with unknown bases or homopolymer runs longer than 6bp. The remaining sequences were denoised and chimeric sequences removed. 16S rRNA gene sequences were clustered into Operational Taxonomic Units (OTUs) at a similarity cutoff value of 97% using the UPARSE algorithm. OTUs were mapped to an optimized version of the SILVA Database^59,60^ containing only the 16S V4 region to determine taxonomies. Abundances were recovered by mapping the demultiplexed reads to the UPARSE OTUs. A rarefied OTU table from the output files generated in the previous two steps was used for downstream analyses of α-diversity, β-diversity,^61^ and phylogenetic trends. Differential taxa abundance was assessed using linear discriminant analysis (LDA) effect size (LEfSe) as previously described.^32^ Fecal 16S sequencing data will be deposited in MG-RAST.

#### Histological staining

Intestinal tissue fragments were fixed in 10% formalin and embedded in paraffin. 7-μm sections were deparaffinized by heating them at 55 to 65°C for 10min, cleared with xylene, and rehydrated through an ethanol gradient to water. For periodic-acid Schiff (PAS) and hematoxylin and eosin (H&E) stainings, standard histological techniques were used. H&E sections of the colon and ileum portions were scored in a blinded fashion by a board certified veterinary pathologist following a modified protocol developed by Dr. Sartor and described elsewhere.^8,62,63^ Criteria for the evaluation included goblet cell number, crypt abscesses, ulceration, mucosa and submucosa mononuclear cellular infiltration. Assessment of the crypt to villi ratio was performed as previously described.^64^ Briefly, H&E photomicrographs from ileal sections were analyzed using Image J software (National Institutes of Health, Bethesda, MD). At least 10 distinct villi and crypts per micrograph, for a total of 3 photomicrographs per mouse (n = 3) were measured and lengths were used to calculate crypt:villi ratios.

#### Quantification of fecal lipocalin

Fecal lipocalin-2 (Lcn-2) analyses were conducted using the mouse Lipocalin-2/NGAL DuoSet ELISA kit (R&D Systems).

#### Genetic characterization of E. coli isolates

Five individual *E. coli* colonies from each biopsy were screened by random amplified polymorphic DNA-polymerase chain reaction (RAPD-PCR) with informative primers 1254 and 1283 ^65^ for genetic diversity. A representative isolate from biopsy sample was selected for further testing. The major *E. coli* phylogenetic groups (A, B1, B2 and D) were determined by triplex PCR.^65^

Representative *E. coli* isolates were screened for the presence of diarrheagenic *E. coli* virulence genes encoding heat labile (LT) and heat stable (STa) toxins, shiga like toxin types I and II (STXI and STXII), intimin-gamma (*eae*), invasion plasmid antigen H (*ipaH*) and enteroaggregative *E. coli* (EAEC) probe pCVD432 as described previously.^66,67^ Strains lacking these genes were screened for the presence of extraintestinal pathogenic *E. coli* and adherent-invasive *E. coli* virulence genes (*ratA*, *pmtI*, *hcp*, *lpfA141*, *lpfA154*, *fyuA*, *chuA*, *kpsMII*, *iss*, *malX*, *gsp*, *traC*, *afaC*, *focG*, *ibeA*, *papC*, *sfa*, *cnf1* and *pduC*) and ColV plasmid specific DNA by PCR.^3,68^

#### Functional characterization of E. coli isolates

Human colonic epithelial cell line Caco-2 (ATCC HTB-37) were grown in minimum essential medium (Gibco, Rockville, MD) supplemented with 15% Fetal Bovine Serum (FBS), 1 mM sodium pyruvate, and 0.1 mM non-essential amino acids solution (NEAA). The murine macrophage-like cell line J774A.1 (ATCC TIB-67) was grown in RPMI 1640 (Gibco-Invitrogen, Grand Island, NY) supplemented with 10% FBS. Monolayers of both cell lines were maintained at 37°C in 5% CO_2_: 95% air (vol/vol). The invasive ability of *E. coli* isolates was evaluated in Caco-2 cells by the gentamicin protection assay as described previously.^37^ Each assay was run in duplicate and repeated three times. Invasion was expressed as the total number of colony forming units (CFU) per ml recovered per well. Non-invasive *E. coli* strains (DH5α and T75) and AIEC strains (541–15 and LF82) were used as negative and positive controls, respectively.

Intracellular survival and replication in J774 macrophages was determined as described previously.^37^ The number of bacteria surviving the gentamicin-killing assay was determined at 1 h and 24 h post-gentamicin treatment. Survival was expressed as the mean percentage of the number of bacteria recovered after 1 h post-infection, defined as 100%.

#### Epithelial cell isolation

Ileal tissue was collected and, after removal of Peyer’s patches, the intestine was opened longitudinally and washed extensively with ice-cold phosphate-buffered saline. The tissue was then cut into 1-cm pieces, which were incubated with 1mM DTT at room temperature with gentle shaking for mucus removal. Tissue was then incubated twice with 30mM EDTA at 37°C for 10min with gentle shaking. The suspension was washed and passed through a 70μm strainer. Cells were stored in RLT buffer (Qiagen).

#### Intestinal immune cell isolation and flow cytometry analyses

Lamina propria mononuclear cells were isolated from ileal tissue as previously described.^40^ For gene expression, cells were stored in RLT buffer (Qiagen). For flow cytometry analysis, LIVE/DEAD fixable aqua dead cell stain kit (Molecular Probes) was used to exclude dead cells. For transcription factor detection, cells were stained for surface markers before fixation and permeabilization with Intracellular Fixation and Permeabilization kit as per manufacturer’s instructions (eBiosciences) for intracellular staining. For cytokine detection, cells were stimulated with phorbol myristate acetate (PMA, 20ng/mL) and ionomycin (1μg/mL) in the presence of BD GolgiPlug for 4 hours before staining. Following surface-marker staining cells were prepared as per manufacturer’s instruction with Cytofix/Cytoperm buffer set (BD Biosciences) for intracellular cytokine evaluation. Data acquisition was computed with BD LSRFortessa flow cytometer and analysis performed with FlowJo software (Tree Star).

#### Bacterial quantification in ileal contents and mucosal scrapings

To assess colony-forming units (CFU), ileal contents or mucosal scrapings were resuspended in sterile phosphate buffered saline (PBS), serially diluted, and plated on Difco^™^ MacConkey agar (BD Biosciences) at 37°C for ~16h. To assess relative abundance, bacterial DNA was isolated from ileal contents or mucosal scrapings using PowerMag Soil DNA Isolation Kit (MO BIO, Carlsbad, CA) following the manufacturer’s instructions. Relative abundance to total bacteria was measured by 16S rRNA quantitative polymerase chain reaction (qPCR).

#### Gene expression by quantitative PCR

Ileal epithelial cells and lamina propria mononuclear cells were isolated from ileal tissue as previously described and stored in RLT buffer (Qiagen). RNA was extracted and purified using RNeasy Plus Mini Kit (Qiagen) and quantified by Nanodrop prior to reverse transcription with iScript cDNA synthesis kit (Bio-Rad). qPCR was performed on an Applied BioSciences Quant Studio 6 Flex Real-time PCR (Applied Biosystems) using PerfeCTa SYBR Green Fast mix, Low ROX (Quanta Biosciences). The thermocycler program was as follows: initial cycle of 95°C for 60 s, followed by 40 PCR cycles at 95°C for 5 s, 60°C for 15 s, 72°C for 15 s. Relative levels of the target genes were determined by calculating the ΔCt to housekeeping gene hprt expression. Primer sequences can be found in Table S3.^20,69–72^

#### *Ex vivo* IL-23 detection

To evaluate IL-23 production in tissue explants, a 1-cm piece of the ileum was collected, thoroughly washed, and cultured overnight in sterile cRPMI (RPMI 1640, 10% FBS, 2.5% Hepes, 1% Sodium pyruvate, 1% L-glutamine, 1% Penicillin/Streptomycin and 50 μM β-mercaptoethanol). IL-23 production was measured in the supernatants using Mouse IL-23 DuoSet ELISA as per manufacturer’s instructions (R&D Systems).

#### ER stress inhibition studies

Mice received 100 μg of ER stress inhibitor 4-Phenylbutyrate (4-PBA) (Sigma-Aldrich, Saint Louis, MO) intraperitoneally every 3 days for a period of 3 weeks.

#### Conditional depletion of intestinal antigen-presenting cells

Mice were treated intraperitoneally every other day with 200ng diphtheria toxin (DT, Sigma-Aldrich) during colonization for selective depletion of CD103^+^ DCs or CX_3_CR1+ MNPs.

#### AIEC growth curve

Starter cultures of AIEC in PYG were sub-cultured in reduced, sterile PYG containing 250mM or 1mM of 4-PBA or equivalent DMSO control. Growth assays were performed in sterile, clear, flat-bottom 96-well plates (Falcon), overlayed with sterile mineral oil, and sealed with an optically clear plate seal (Microseal ‘B’, BioRad). Growth was measured every 15 minutes by absorbance, OD_600_, on a Tecan Infinite F50 microplate reader with shaking at 7.8 Hz between readings. Growth assays were conducted at 37°C in the anaerobic chamber.

#### Organoid generation, maintenance and infection

##### Organoid generation

Organoid isolation was performed as previously described.^73^ Briefly, 15 cm of the proximal small intestine was removed, flushed, and washed with cold PBS. The intestine was cut longitudinally and villi were scraped using a hemacytometer coverslip leaving the crypts attached. The tissue was then cut into 5-mm pieces and placed into 10 mL cold PBS and vigorously resuspended with a 10mL pipette. The supernatant was aspirated and fresh PBS was added ~20X until the supernatant was clear. 25 mL 5mM EDTA-PBS was added and incubated while shaking at 4°C for 30 min. The supernatant was aspirated, 10 mL of cold PBS was added, and samples were vigorously resuspended and passed through a 70-μm strainer. This procedure was repeated 3–5 times, each successive fraction was collected and examined under a microscope for the presence of intact intestinal crypts and the absence of villi. Crypt fractions were centrifuged at 800 rpm for 5min and then mixed with 10 mL of IntestiCult organoid growth media and centrifuged again at 600rpm for 2min to remove single cells. Samples were then filtered through a 70-μm filter into an FBS (1 mL)-coated tube and spun at 1,200 rpm for 3 min. The supernatant was aspirated, and the cell pellets (purified crypts) were resuspended in Growth Factor Reduced (GFR) Matrigel, and plated in multiple wells of a 48-well plate. After polymerization for 15 min at 37°C, 250 μL of IntestiCult organoid growth media (basal medium containing 50 ng/mL EGF, 100 ng/mL Noggin, and 1 μg/ml R-spondin) was then laid on top of the GFR Matrigel.

##### Organoid maintenance

The medium was changed every 2 days, and organoids were passaged 1:4 every 7 days. For passaging, the growth medium was removed, and the Matrigel was resuspended in Cell Recovery Solution (Corning). The organoids were mechanically disassociated by pipetting 50–100 times and shaking at 4°C for 20min. 7 mL of cold PBS was added to the tube and pipetted 20 times to fully wash the cells. The cells were then centrifuged at 2,000 rpm for 5 min, and the supernatant was aspirated. Cells were then resuspended in GFR Matrigel and replated as described above.

##### Organoid infection

Crypt fragments were infected during passaging. After release from GFR Matrigel, organoids were mechanically shredded into fragments by pipetting, incubated with either non-pathogenic T75 or AIEC MSL1 for 1h and re-embedded in GFR Matrigel for new organoid formation. Stimulation with 10μM tunicamycin was used as a positive control for ER stress induction. Cells were harvested at 48h post-infection and stored in RLT buffer (Qiagen).

#### ER stress induction and infection in MC38 cells

MC38 epithelial cells were grown in RPMI 1640 supplemented with 10% FBS, 1% Penicillin/Streptomycin and 1% L-glutamine. MC38 epithelial cells were pre-treated with 1μM tunicamycin for 4h to induce ER stress prior to infection with AIEC MSL1 or non-pathogenic T75 control at multiplicity of infection (MOI) 20. 1h post-infection, cells were washed and cultured in MC38 media supplemented with 20ng/mL gentamicin for 24h. CFU enumeration was performed by lysing cells in 1% Triton X-100/PBS. Lysates were serially diluted in PBS and plated on Luria-Bertani (LB) agar at 37°C for ~16h.

#### Generation of iCD103-DCs from bone marrow

Bone marrow (BM) was flushed out from sterilized femur and tibia of euthanized mice. Red blood cells were lysed using ACK Lysis buffer (Gibco). iCD103-DCs cells were generated as previously described.^42^ Briefly, 15 × 10^6^ BM cells were cultured in 10mL of RPMI 1640 medium supplemented with 10% heat-inactivated FBS, 1% Penicillin/Streptomycin, 50 μM β-mercaptoethanol, 200ng/mL recombinant murine FLT-3 Ligand (FLT3L) (14–8001, eBioscience) and 5ng/mL recombinant murine granulocyte macrophage colony-stimulating factor (GM-CSF) (315–03, Peprotech). 5mL complete medium was added between days 5 and 6 to minimize apoptosis. Non-adherent cells were harvested on day 9, counted and re-plated at 3 × 10^6^ cells in 10 mL complete medium supplemented with FLT3L and GM-CSF as on day 0. Non-adherent iCD103-DCs were harvested on day 15 and used for experiments.

##### *In vitro* co-culture

MC38 cells were treated with 10μM tunicamycin for 1h to induce ER stress. Cells were then washed and cultured for 4h with complete RPMI 1640 media. MC38 supernatants were collected and used to stimulate BM-induced CD103^+^-DCs in the presence or absence of 200ng/mL flagellin for 24h. IL-23 production was measured by ELISA as described above.

### QUANTIFICATION AND STATISTICAL ANALYSES

Plots were built in GraphPad Prism (San Diego, CA) and R studio (Boston, MA). Boxplots present the median, 25^th^ and 75^th^ percentiles and bar as well as scatter plots present mean ± standard error of the mean (SEM). For comparison of two groups, significance of categorical variables such as gene expression, cytokine or lipocalin levels quantification, cell populations, histology score and bacterial loads were assessed by Student’s t or Mann-Whitney test. For comparison of more than two groups, Neuman-Keuls multiple comparison test was used. Epithelial cell invasion and survival in macrophages were analyzed by Kruskall-Wallis with Dunnett’s multiple comparisons test. For weight loss analysis repeated measures ANOVA was applied. Significance for survival curves were analyzed using a Kaplan-Meier Log-Ranked test. Hypothesis testing was done using two- sided test as appropriate at a 95% significance level. A value of p < 0.05 was considered statistically significant.

## Supplementary Material

1

## Figures and Tables

**Table:** KEY RESOURCES TABLE

REAGENT or RESOURCE	SOURCE	IDENTIFIER
Antibodies		

Live/Dead cell stain kit	ThermoFisher	Cat# L34962
CD3-eFluor780 (clone ID UCHT1)	ThermoFisher	Cat# 47–0038-42; RRID: AB_1272042
CD3-FITC (clone ID 145–2C11)	ThermoFisher	Cat# 11–0031-63; RRID: AB_464880
CD4-AlexaFluor700 (clone ID GK1.5)	ThermoFisher	Cat# 54–0041-80; RRID: AB_494001
CD4-eFluor780 (clone ID RM4–5)	ThermoFisher	Cat# 47–0042-80; RRID: AB_1272219
IL-17-PE (clone ID B2D)	ThermoFisher	Cat# 12–7177-81; RRID: AB_763582
IFNγ-PE-Cy7 (clone ID XMG1.2)	ThermoFisher	Cat# 25–7311-41; RRID: AB_1257211
T-bet-e660 (clone ID eBio4B10)	ThermoFisher	Cat# 50–5825-82; RRID: AB_10596655
RORgt-PE (clone ID B2D)	ThermoFisher	Cat# 12–6981-80; RRID: AB_10805392
GATA3-BUV395 (clone ID L50–823)	BD Biosciences	Cat# 565448; RRID: AB_2813884
Foxp3-e450 (clone ID FJK-16 s)	ThermoFisher	Cat# 48–5773-80; RRID: AB_1518813

Bacterial and virus strains		

AIEC MSL1 (isolated from *Agr2-knockout* mice)	This work	N/A
AIEC MSL6 (isolated from *Agr2-knockout* mice)	This work	N/A
Non-AIEC *E. coli* isolate T75 (isolated from CD patient)	Baumgart et al.^37^	N/A
AIEC CUMT8	Dogan et al.^3^	N/A
AIEC CUMT8^Δ*lpfA154*^	Dogan et al.^3^	N/A
*Bacteroides thetaiotaomicron*	Benjdia et al.^55^	N/A

Chemicals, peptides, and recombinant proteins		

Phorbol myristate acetate	Sigma-Aldrich	Cat# P1585
Ionomycin calcium salt	Sigma-Aldrich	Cat# I0634
GolgiPlug	BD Bioscience	Cat# 51–2301KZ
SYBR Green Supermix	Roche	Cat# 4887352001
0.5M EDTA pH8.0	Invitrogen	Cat# AM9261
DL-Dithiothreitol	Sigma-Aldrich	Cat# D9779
RPMI without L-glutamine	GE Healthcare	Cat# SH30096.01
DMEM, High Glucose, GlutaMAX	Gibco	Cat# 10–566-016
Supplement		
IntestiCult Organoid growth media (Mouse)	STEMCELL Technologies	Cat# 06005
Cell Recovery Solution	Corning	Cat# 354253
Matrigel Matrix	BD Bioscience	Cat# 354234
Sodium pyruvate	Sigma-Aldrich	Cat# S8636
L-glutamine	Corning Life Sciences	Cat# 25–005-Cl
Penicillin/Streptomycin	HyClone	Cat# SV30010
2-mercaptoethanol	Gibco	Cat# 21985–023
HEPES	HyClone	Cat# SH30237.01
Collagenase 8	Sigma-Aldrich	Cat# C2139
Deoxyribonuclease I from bovine pancreas	Sigma-Aldrich	Cat# DN25
Percoll	GE Health	Cat# 17–0891-01
Formalin	Sigma-Aldrich	Cat# 65346
Sodium 4-Phenylbutyrate	Sigma-Aldrich	Cat # SML0309
Gentamicin	ThermoFisher	Cat# 15750060
Tunicamycin	Sigma-Aldrich	Cat# SML1287
Flagellin	ProSpec	Cat# PRO-1240
Mouse FLT3 Ligand recombinant protein	ThermoFisher	Cat# RP-8665
Recombinant murine GM-CSF	PeproTech	Cat# 315–03
Recombinant mouse R-Spondin 1 Protein	R&D Systems	Cat# 3474-RS-050
Recombinant murine Noggin	PeproTech	Cat# 250–38
Recombinant murine EGF	PeproTech	Cat# 315–09
Peptone Yeast Glucose Broth	Anaerobe Systems	Cat# AS-822
MacConkey Agar	Sigma-Aldrich	Cat# M7408
Luria-Bertani Agar	BD	Cat# 244520

Critical commercial assays		

Mouse Lipocalin-2/NGAL DuoSet ELISA	R&D Systems	Cat# DY1857
Mouse IL-23 DuoSet ELISA	R&D Systems	Cat# DY188705
RNeasy Plus Mini Kit	QIAGEN	Cat# 74134
RNeasy Plus Micro Kit	QIAGEN	Cat# 74034
iScript cDNA synthesis kit	Bio-Rad	Cat# 1708891
FoxP3/Transcription Factor Staining Buffer set	eBioscience	Cat# 00–5523-00
Fixation/Permeabilization Concentrate	ThermoFisher	Cat# 00–5123-43
Fixation/Permeabilization Diluent	ThermoFisher	Cat# 00–5223-56
DNeasy PowerLyzer PowerSoil Kit	Qiagen	Cat# 12855

Deposited data		

Agr2 microbiome 16S sequencing data	NCBI SRA	BioProject ID PRJNA886765

Experimental models: organism/strain		

Mouse: C57BL/6	Jackson Laboratories	Cat# 000664
Mouse: *Agr2-knockout*	Zhao et al.^20^	N/A
Mouse: *il23r*-GFP	Awasthi et al.^56^	N/A
Mouse: *Itgax-Cre*	Jackson Laboratories	Cat# 008068
Mouse: *Cd207-DTR/GFP*	Jackson Laboratories	Cat# 016940
Mouse: *Cx3cr1*^*tm3(DTR)Litt*^/J	Jackson Laboratories	Cat# 025629
Cell line: Caco-2	ATCC	Cat# HTB-37
Cell line: J774A.1	ATCC	Cat# TIB-67
Cell line: MC38	Dr. Greg Sonnenberg, WCM	N/A

Oligonucleotides		

Eubacteria probe: EUB-338; GCTGCCTCCCGTAGGAGT	Simpson et al.^57^	N/A
*E. coli/Shigella* probe: *E. coli* 16S rRNA; GCAAAGGTATTAACTTTACTCCC	Simpson et al.^57^	N/A
See Table S3 for primer sequences	N/A	N/A

Software and algorithms		

R	R Core Team	N/A
GraphPad Prism 7	GraphPad Software	N/A
FlowJo LLC v8.7	Becton Dickenson	N/A

## Highlights

- AGR2 is associated with *Enterobacteriaceae* dysbiosis in Crohn’s disease
- AGR2-dependent epithelial ER stress leads to the expansion of mucosa-associated AIEC
- AIEC are sufficient to induce ER-stress-dependent Th17 ileocolitis
- Epithelial ER stress triggers CD103^+^ DC production of IL-23 required for ileocolitis
