# Supplementary material for: Agr2-associated ER stress promotes adherent-invasive E. coli dysbiosis and triggers CD103+ dendritic cell IL-23-dependent ileocolitis
Source: Cell Rep. Author manuscript; Available in PMC 2023 Jan 1. (PMC9805753; doi:10.1016/j.celrep.2022.111637)
Supplement: 1 [file NIHMS1850755-supplement-1.pdf]

Cell Reports, Volume *41*

Supplemental information

Agr2-associated ER stress promotes adherent-invasive *E. coli* dysbiosis and triggers CD103^{+} dendritic cell IL-23-dependent ileocolitis

Monica Viladomiu, Manirath Khounlotham, Belgin Dogan, Svetlana F. Lima, Ahmed Elsaadi, Emre Cardakli, Jim G. Castellanos, Charles Ng, Jeremy Herzog, Alexi A. Schoenborn, Melissa Ellermann, Bo Liu, Shiying Zhang, Ajay S. Gulati, R. Balfour Sartor, Kenneth W. Simpson, Steven M. Lipkin, and Randy S. Longman

Supplemental tables

Factor	Df	SumsOfSqs	MeanSqs	F.Model	R^2	Pr(>F)
**AGR2**	1	0.708	0.708	2.599	0.014	**0.006**
**Diagnosis**	1	1.006	1.006	3.691	0.020	**0.001**
Gender	1	0.283	0.283	1.039	0.006	0.373
Age	1	0.167	0.167	0.615	0.003	0.876
AGR2:Diagnosis	1	0.194	0.194	0.711	0.004	0.784
Residuals	173	47.140	0.272			
Total	178	49.498				

Table S1. Impact of AGR2 expression, disease, and demographics on β-diversity of IBD fecal microbiome. ***Related to Figure 1.*** Stool 16S sequencing data and matching RNA sequencing from ileal mucosal biopsies was evaluated for non-IBD (n=50) and CD (n=253) patients from the RISK Cohort Study. β-diversity correlations to AGR2 expression levels, diagnosis, gender and age were calculated using Adonis.

Correlation	Spearman correlation coefficient (r)	Standard error	p-val	q-val
AGR2 & Erysipelotrichaceae	-0.00382	0.00147	0.00993	0.0674
AGR2 & Bacteroidaceae	-0.028	0.0116	0.01685	0.0674
AGR2 & Porphyromonadaceae	-0.00331	0.00136	0.01595	0.0674
AGR2 & Pasteurellaceae	0.0153	0.0073	0.03751	0.11252
AGR2 & Enterobacteriaceae	0.02361	0.01221	0.05476	0.13142
AGR2 & Desulfovibrionaceae	-0.00158	0.0009	0.08098	0.16195

*q-val < 0.2

Table S2. *AGR2* correlation with 16S at a family level. *Related to Figure 1.* Stool 16S sequencing data and matching RNA sequencing from ileal mucosal biopsies of non-IBD and CD patients from the RISK cohort. Spearman correlation between mucosal *AGR2* expression and stool family relative abundance is shown for q-val < 0.2. Positive correlation in orange and negative correlation in green.

**Primer sequence**	**Reference**
il23p19 F: 5'-CCAGCAGCTCTCTCGGAATC-3'<br>*il23p19 R*: 5'-GATTCATATGTCCCGCTGGTG-3'	(71)
Chop F: 5’-CTGGAAGCCTGGTATGAGGAT-3’<br>*Chop R*: 5’- CAGGGTCAAGAGTAGTGAAGGT-3’	(20)
Perk F: 5’- GCTGGCGAACCGGAGTCACG-3’<br>*Perk R*: 5’- CCAGTGCAGCGATTCGTCCA-3’	(20)
Gpr78 F: 5′-CATGGTTCTCACTAAAATGAAAGG-3′<br>*Gpr78 R:* 5′-GCTGGTACAGTAACAACTG-3′	(73)
Spliced *Xbp1 F:* 5′-GAGTCCGCAGCAGGTG-3′<br>Spliced *Xbp1 R:* 5′-GTGTCAGAGTC-CATGGGA-3′	(73)
Total *Xbp1 F:* 5′-AAGAACACGCTTGGGAATGG-3′<br>Total *Xbp1 R:* 5′-ACTCCCCTTGGCCTCCAC-3′	(73)
Hprt F: 5′-GAGGAGTCCTGTTGATGTTGCCAG-3′<br>*Hprt R:* 5′-GGCTGGCCTATAGGCTCATAGTGC-3′	(72)
Universal 16S F: UniF340 5’-ACTCCTACGGGAGGCAGCAGT-3’<br>Universal 16S R: UniR514 5’-ATTACCGCGGCTGCTGGC-3’	(70)
Enterobactericieae F: Uni515F 5’-GTGCCAGCMGCCGCGGTAA-3’<br>Enterobactericieae R: Ent826R 5’-GCCTCAAGGGCACAACCTCCAAG-3’	(70)
Bacteroides F: BactF285 5’-GGTTCTGAGAGGAGGTCCC-3’<br>Bacteroides R: UniR338 5’-GCTGCCTCCCGTAGGAGT-3’	(70)
E. coli F: 5’-GGTAGAGCACTGTTTTGGCA-3’<br>E. coli R: 5’-TGTCTCCCGTGATAACTTTCTC-3’	(70)

Table S3. Primer sequences. ***Related to Key Resources Table.***

Figure S1

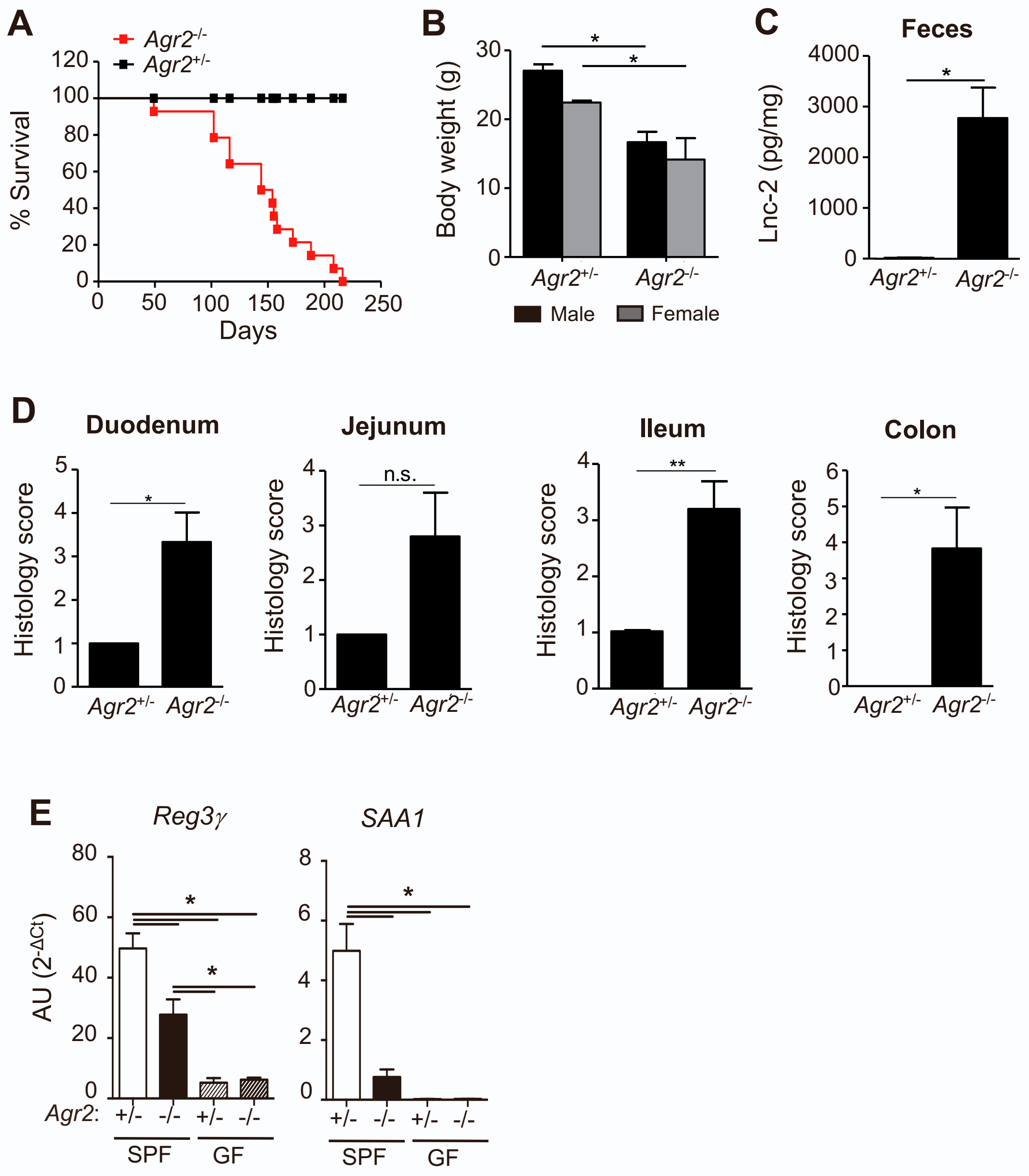

FIGURE S1. SPF *Agr2*$^{-/-}$ mice develop spontaneous ileocolitis. *Related to Figure 2.* A. SPF littermate *Agr2*$^{+/-}$ and *Agr2*$^{-/-}$ mice were monitored for survival for 6 months. **B, C.** Body weight (**B**) and fecal lipocalin-2 levels (**C**) at 6 months. **D.** Duodenum, jejunum, ileum and colon were stained with H&E for histology evaluation. Representative data of two independent experiments are shown as mean ± SEM. *p<0.05, **p<0.02, t-test. **E.** SPF and GF *Agr2*$^{+/-}$ and *Agr2*$^{-/-}$ mice were monitored for 6 months. Expression of *Reg3g* and *Saa1* was measured in ileal epithelial cells by quantitative PCR**.** n=4 from one of three experiments. Error bars represent SEM. *p<0.05, t-test.

Figure S2

A

Group	Strain ID	Serotype	Phylo group	pduC	pks	chuA	iroN	ratA	Pmtl	colV	hcp	lpfA141	lpfA154	kpsMII	fyuA	iss	malX	GSP	VIRB	afaBC	focG	ibeA	papC	sfaDE	LT	Sta	STb	stx1	stx2	eae	cnf1
Agr2$^{-/-}$ mice AIEC	MSL1	O2:H(6 or 41)	B2	-	+	+	+	+	-	+	-	-	-	+	+	-	+	+	-	-	-	-	-	+	-	-	-	-	-	-	-
	MSL6	O2:H(6 or 41)	B2	-	+	+	+	+	-	+	-	-	-	+	+	-	+	+	-	-	-	-	-	+	-	-	-	-	-	-	-
Reference AIEC	NC101	O2:H(6 or 41)	B2	+	+	+	+	+	-	+	-	-	-	+	+	-	+	+	-	-	-	+	-	+	-	-	-	-	-	-	-
	LF82	O83:H1	B2	+	-	+	-	+	+	+	-	+	-	+	+	-	+	+	-	-	-	+	-	-	-	-	-	-	-	-	-
	CUMT8	O8:H21	B1	+	-	-	-	-	-	-	+	-	+	-	-	-	-	+	-	-	-	-	-	-	-	-	-	-	-	-	-
	541-15	O21:H33	A	-	-	-	-	-	-	-	+	-	+	-	+	-	-	+	-	-	-	-	-	-	-	-	-	-	-	-	-
Non-AIEC *E. coli*	T75	O39:H10	A	-	-	-	-	-	-	-	+	-	-	-	-	-	-	+	-	-	-	-	-	-	-	-	-	-	-	-	-
	Dh5α	OR:H48	A	-	-	-	-	-	-	-	-	-	-	-	-	-	-	+	-	-	-	-	-	-	-	-	-	-	-	-	-

(Virulence Genes: pduC through cnf1)

B

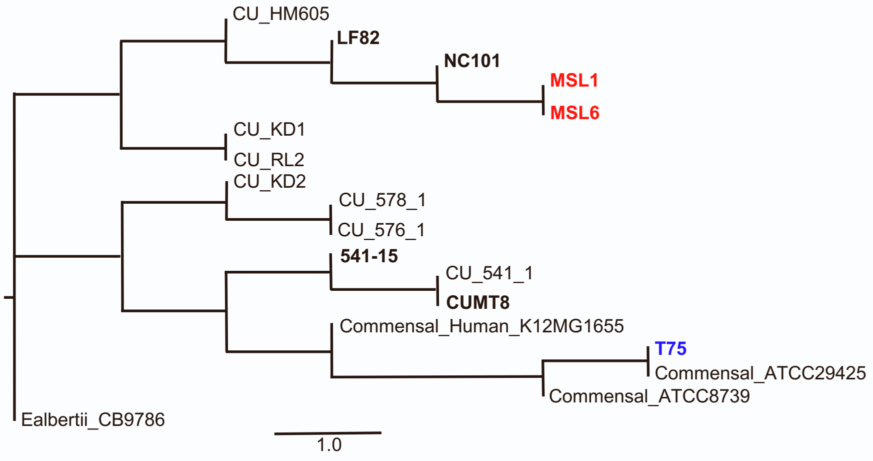

FIGURE S2. Characterization of mucosa-associated bacteria and adherent-invasive *E. coli* isolates from SPF *Agr2*$^{-/-}$ mice. *Related to Figure 2.* A. Genetic characterization of *Agr2*$^{-/-}$ mice-derived MSL1 and MSL6 AIEC strains in comparison to human AIEC isolates LF82 and 541-15, murine AIEC strains NC101 and CUMT8 and non-pathogenic T75 and DH5α. Multiplex PCR was performed for *E. coli* and AIEC-associated virulence factors. **B.** Phylogenetic tree depicting the relationship between MSL1 and MSL6 and other known human and murine AIEC.

Figure S3

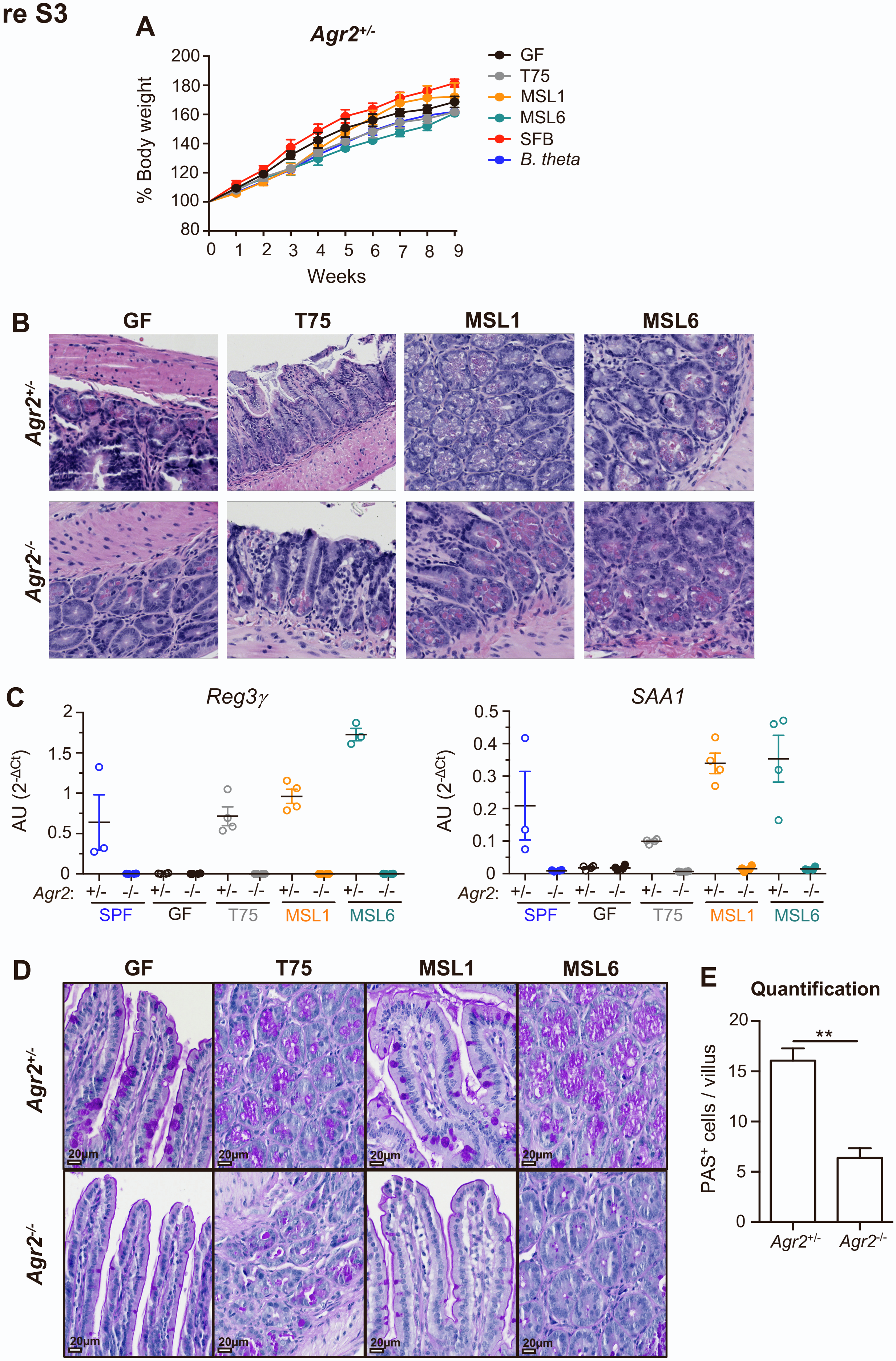
A
Agr2+/-
% Body weight
Weeks
GF
T75
MSL1
MSL6
SFB
B. theta
B
GF
T75
MSL1
MSL6
Agr2+/-
Agr2-/-
C
Reg3γ
SAA1
AU (2-ΔCt)
Agr2:
+/-
-/-
SPF
GF
T75
MSL1
MSL6
D
GF
T75
MSL1
MSL6
Agr2+/-
Agr2-/-
20μm
E
Quantification
PAS+ cells / villus
**
Agr2+/-
Agr2-/-

FIGURE S3. Paneth and goblet cell characterization in *Agr2*$^{-/-}$ mice. ***Related to Figure 2.*** **A-D.** GF *Agr2*$^{+/-}$ and *Agr2*$^{-/-}$ mice were monocolonized at weaning with non-pathogenic T75, AIEC MSL1, AIEC MSL6, SFB or *Bacteroides thetaiotaomicron* for 9 weeks. Mice were monitored for body weight (**A**). Ileal sections were stained with H&E for histology evaluation (**B**)**.** Expression of *Reg3γ* and *Saa1* was measured in ileal epithelial cells by quantitative PCR (**C**)**.** Ileal sections were stained with periodic-acid Schiff (PAS) stain for goblet cell evaluation (**D**). Number of PAS^{+} cells per villus were quantified (**E**). n=3-5 from one of four experiments. Error bars represent SEM. *p<0.05, ANOVA.

Figure S4

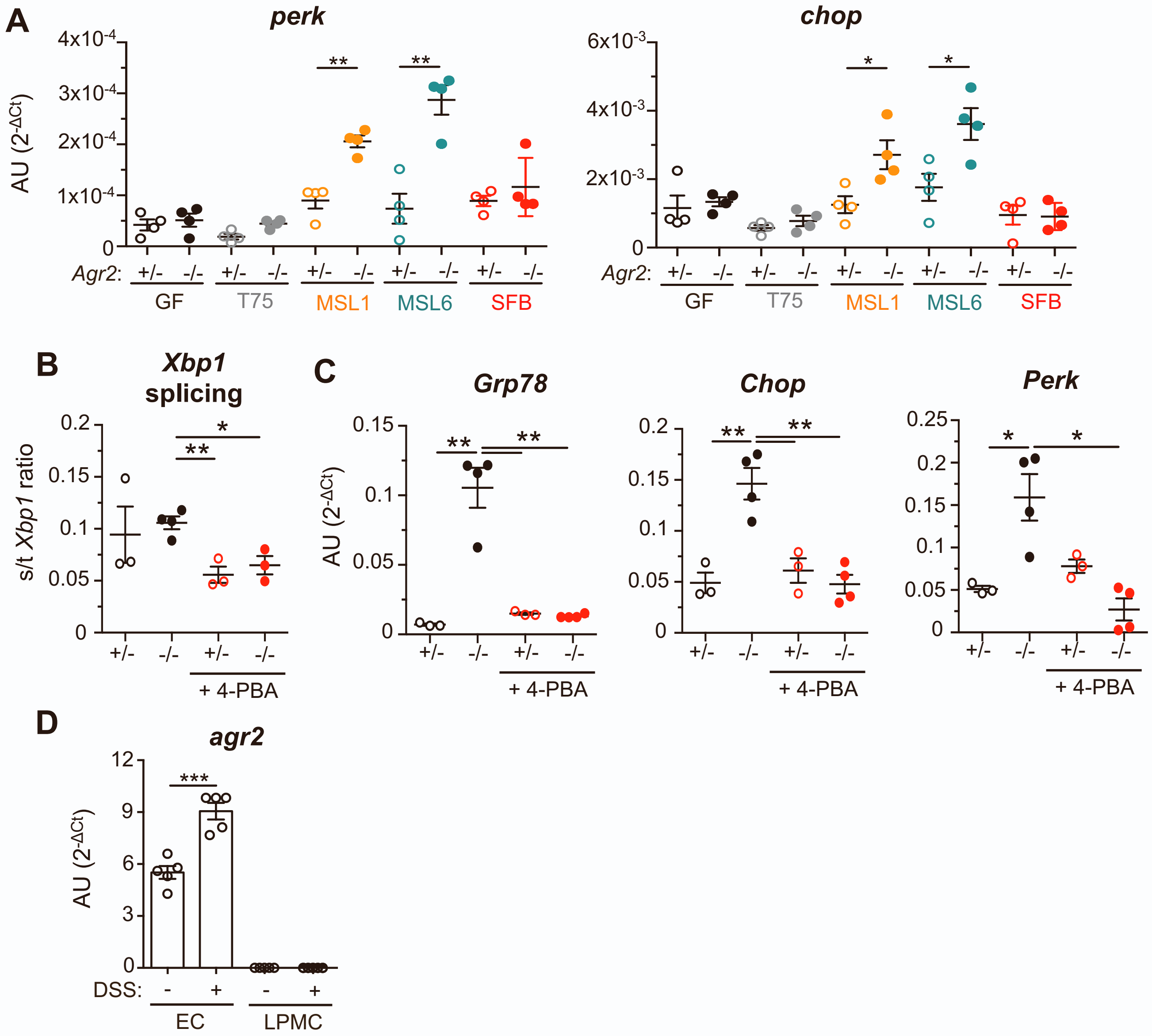

FIGURE S4. AIEC induce epithelial ER stress in gnotobiotic *Agr2*$^{-/-}$ mice. *Related to Figure 3.* **A.** GF *Agr2*$^{+/-}$ and *Agr2*$^{-/-}$ mice were monocolonized at weaning with non-pathogenic T75, AIEC MSL1, AIEC MSL6 or SFB for 10 weeks. Expression of *perk* and *chop* was measured in ileal epithelial cells by quantitative PCR**.** n=4-5 from one of four experiments. Error bars represent SEM. **p*<0.05, ***p*<0.01, ANOVA. **B.** GF *Agr2*$^{+/-}$ and *Agr2*$^{-/-}$ mice were monocolonized at weaning with AIEC MSL1 for 3 weeks. Mice were treated with the ER stress inhibitor 4-phenylbutyrate (4-PBA) or PBS control every 3 days. *Xbp1* splicing (**B**) and expression of *Grp78, Perk* and *Chop* (**C**) was measured in ileal epithelial cells by quantitative PCR. n=3-4 mice per group from one of two experiments. Error bars represent SEM. **p*<0.05, ***p*<0.01, ANOVA. . **D.** SPF C57BL/6 WT mice we treated with 2% dextran sodium sulfate in the drinking water *ad libitum* for 7 days. Expression of *agr2* was measured in colonic epithelial cells and lamina propria mononuclear phagocytes by quantitative PCR**.** n=5. Error bars represent SEM. ****p*<0.005, ANOVA.

Figure S5

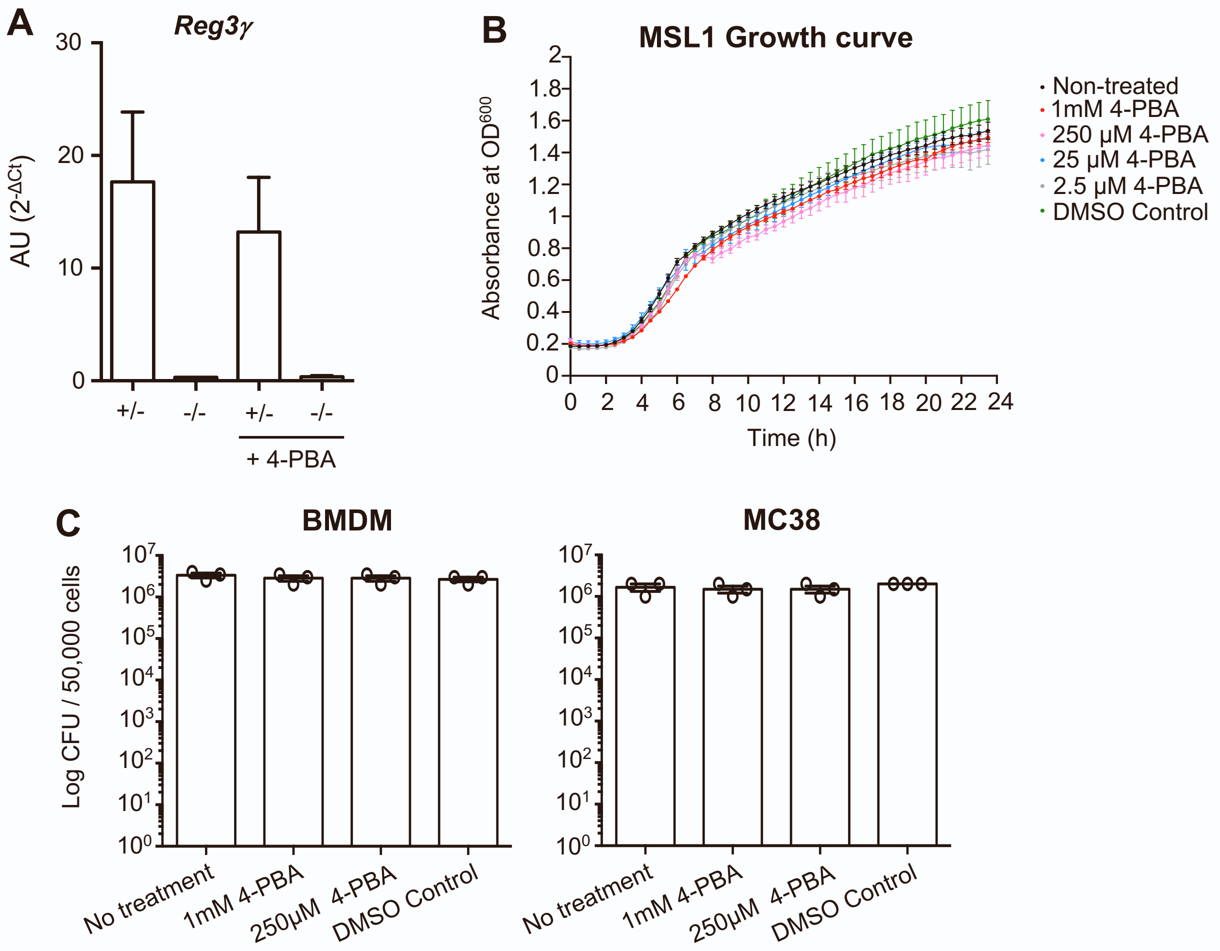

FIGURE S5. Effect of 4-PBA treatment on AIEC growth. *Related to Figure 4.* **A.** GF *Agr2*$^{+/-}$ and *Agr2*$^{-/-}$ mice were monocolonized at weaning with AIEC MSL1 for 3 weeks. Mice were treated with the ER stress inhibitor 4-phenylbutyrate (4-PBA) or PBS control every 3 days. Expression of *Reg3γ* was measured in ileal LPMCs by quantitative PCR. n=3. Error bars represent SEM. *p<0.05, ANOVA **B, C.** AIEC MSL1 was grown anaerobically in PYG media supplemented with 1mM or 250μM of 4-PBA and monitored for 24 hours. Non-supplemented and DMSO-supplemented media were used as controls (**B**). 4-PBA-treated and non-treated bacteria were then used to infect bone marrow-derived macrophages (BMDM) or MC38 epithelial cells at MOI 20 for 30min. Cells were then washed and cultured with complete media containing 100μg/mL gentamicin for 1h to eliminate extracellular bacteria, and then washed and cultured with complete media containing 20μg/mL gentamicin for 24h. Cells were harvested and lysed with 1% triton for 10min, followed by serial dilution plating in LB agar plates for bacterial re-isolation (**C**). n=3 per group from one of three experiments. Error bars represent SEM. *p<0.05, ANOVA.

Figure S6

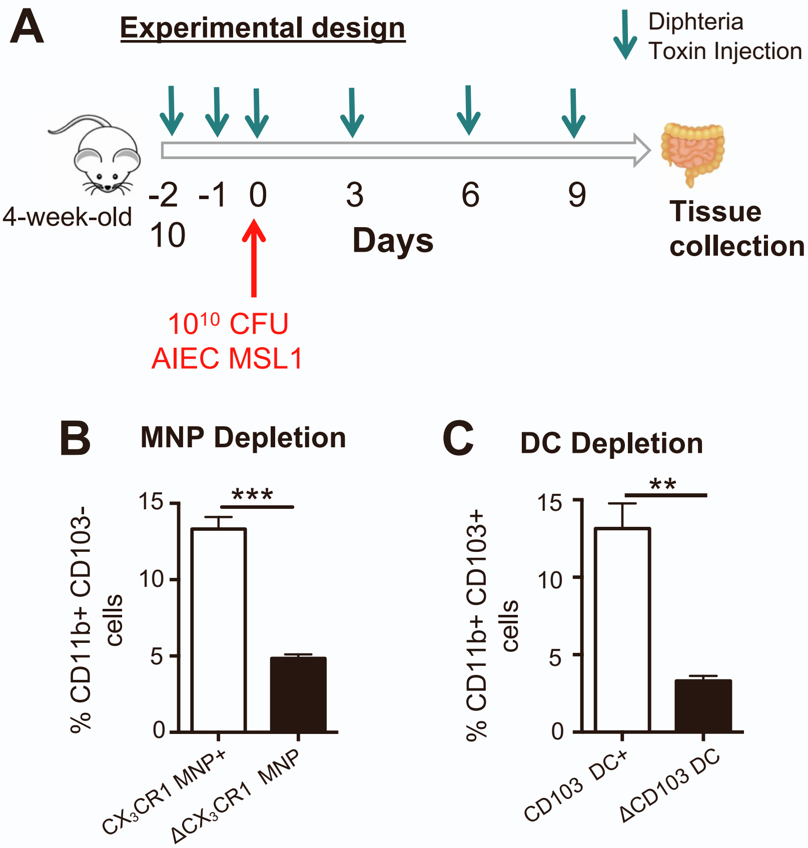

FIGURE S6. Antigen presenting cell depletion strategy*. Related to Figure 7.* **A.** 4-week-old littermate *Cx3cr1-LSL-DTR* (labeled CX_3CR1 MNP+) and *Itgax-cre Cx3cr1-LSL-DTR* (labeled ΔCX3CR1 MNP) mice were treated with diphtheria toxin (DT) before and during colonization with 10^{10} CFU CD-derived AIEC MSL1. 4-week-old littermate *Lang-DTR-EGFP* mice were treated with DT (labeled ΔCD103 DC) or PBS (labeled CD103 DC+) before and during colonization with AIEC MSL1. **B, C.** Flow cytometry of ileal live, $CD11c^+MHCII^+$ cells was used to evaluate depletion of $CD11b^+CD103^-$ (MNPs) (**B**) and $CD11b^+CD103^+$ (DCs) (**C**). n=5 from one of two experiments. Error bars represent SEM. ***p*<0.01, ****p*<0.005 t-test.
